# A Hierarchical Deep Learning Architecture for Diagnosing Retinal Diseases Using Cross-Modal OCT to Fundus Translation in the Lack of Paired Data

**DOI:** 10.3390/jimaging12010036

**Published:** 2026-01-08

**Authors:** Ekaterina A. Lopukhova, Gulnaz M. Idrisova, Timur R. Mukhamadeev, Grigory S. Voronkov, Ruslan V. Kutluyarov, Elizaveta P. Topolskaya

**Affiliations:** 1 Research Laboratory “Sensor Systems Based on Integrated Photonics Devices”, Ufa University of Science and Technology, 32 Z. Validi Street, 450076 Ufa, Russia; voronkov.gs@ugatu.su (G.S.V.); kutluyarov.rv@ugatu.su (R.V.K.); grakhova.ep@ugatu.su (E.P.T.); 2 Department of Ophthalmology, Bashkir State Medical University, 3 Lenin Street, 450008 Ufa, Russia; idguma@mail.ru (G.M.I.); photobgmu@gmail.com (T.R.M.)

**Keywords:** optical coherence tomography, diabetic retinopathy, age-related macular degeneration, diabetic macular edema, hierarchical neural networks, cross-modal learning, multi-label classification, contrastive learning, ophthalmology, computer-aided diagnosis

## Abstract

The paper focuses on automated diagnosis of retinal diseases, particularly Age-related Macular Degeneration (AMD) and diabetic retinopathy (DR), using optical coherence tomography (OCT), while addressing three key challenges: disease comorbidity, severe class imbalance, and the lack of strictly paired OCT and fundus data. We propose a hierarchical modular deep learning system designed for multi-label OCT screening with conditional routing to specialized staging modules. To enable DR staging when fundus images are unavailable, we use cross-modal alignment between OCT and fundus representations. This approach involves training a latent bridge that projects OCT embeddings into the fundus feature space. We enhance clinical reliability through per-class threshold calibration and implement quality control checks for OCT-only DR staging. Experiments demonstrate robust multi-label performance (macro-F1 =0.989±0.006 after per-class threshold calibration) and reliable calibration (ECE =2.1±0.4%), and OCT-only DR staging is feasible in 96.1% of cases that meet the quality control criterion.

## 1. Introduction

Diabetic retinopathy (DR), including diabetic macular edema (DME), and age-related macular degeneration (AMD) are the leading causes of blindness and irreversible vision loss worldwide [1,2,3,4,5]. Currently, DR affects over 100 million people globally, with DME developing in 6–7% of individuals with diabetes mellitus (DM) [6,7]. As of 2021, the global prevalence of DR among patients with DM is 22.27% [6].

In turn, AMD is the leading cause of central vision loss in individuals aged 50 and older in developed countries [8,9]. Additionally, clinical observations reveal a high incidence of co-occurring diseases; for instance, the presence of DR significantly increases the risk of developing AMD [10]. DME can occur at any stage of DR, including the proliferative stage (PDR). In specialized tertiary-level clinical cohorts, the prevalence of DME can reach approximately 20–30% [11,12].

In modern ophthalmology, Optical Coherence Tomography (OCT) is a crucial method for the morphological assessment of macular structures. It provides high-resolution images at the micrometer level, allowing for detailed visualization of the intraretinal architecture [13,14,15,16].

Recent advances in artificial intelligence (AI) for medical image analysis are rapidly reshaping retinal imaging workflows, not only improving automated detection and staging but also enabling clinically meaningful biomarker discovery [17]. In particular, explainable AI (XAI) and the interplay between handcrafted image biomarkers and deep learned features have become central for trustworthy clinical translation and for linking model decisions to interpretable retinal patterns [17]. Moreover, AI-enhanced retinal imaging is increasingly studied as a biomarker for systemic diseases, highlighting that retinal structure and vasculature can serve as a proxy for broader health status beyond ophthalmology [18]. These developments complement OCT-based biomarker studies [15] and further motivate cross-modal and calibrated AI pipelines that remain reliable under real-world constraints such as missing modalities and domain shifts.

On the other hand, color fundus photography is considered the “gold standard” for staging DR in international classifications, such as the Early Treatment Diabetic Retinopathy Study (ETDRS) and the International Clinical Classification of Diabetic Retinopathy (ICDR) [19,20,21,22]. However, this specialization presents a practical challenge: a comprehensive diagnosis requires both OCT and fundus images of the same eye. In actual clinical practice, obtaining both types of images can be difficult due to technical, logistical, and financial constraints [22,23].

Current methods for automatically diagnosing retinal diseases using various imaging modalities encounter several significant limitations. For instance, traditional classification techniques that rely on the softmax function impose an artificial mutual exclusivity among classes. It is insufficient for accurately representing the comorbidity of DR, including DME and its combinations with AMD [24,25]. An alternative approach is to use a multi-target or multi-label formulation with independent sigmoid activations, which allows for a better representation of multiple diseases occurring simultaneously. However, this approach necessitates specialized loss functions and calibration [26,27,28,29]. Moreover, most modern methods depend on the availability of strictly paired OCT and fundus images for both training and testing [30,31,32]. Existing cross-modal methods exhibit significant performance degradation when the corresponding images are unavailable [30,33,34,35,36].

An additional limitation is the class imbalance and domain shifts present in medical data, which result in systematic underrepresentation of rare but clinically significant conditions [37,38,39]. At the same time, variations in scanning parameters across different equipment manufacturers, such as Optovue, Zeiss, and Heidelberg, can lead to domain shifts that negatively impact algorithm performance when transitioning between scanners [40,41,42,43].

Monolithic Convolutional Neural Network (CNN) architectures often demonstrate poor probability calibration [44,45]. It means that even when the overall accuracy (measured by the Brier Score) reaches clinically significant levels due to specific calibration efforts, the Expected Calibration Error—a metric that assesses how well probabilities align with actual outcomes—can still be relatively high. In fact, this error may fluctuate by as much as 4–6% or more [44,45,46].

Traditional architectures that rely on single modalities, such as CNNs like ResNet, EfficientNet, and ConvNeXt, as well as Vision Transformers such as ViT and Swin Transformer [47,48,49,50], have limitations. They cannot leverage complementary information across modalities and may exhibit inconsistent performance when faced with domain shifts [51,52,53,54,55,56]. Recent hybrid frameworks that combine CNNs and Transformers demonstrate that merging the local inductive bias of CNNs with the global attention mechanism of Transformers can create a robust baseline for medical image classification and reporting [57].

Current cross-modal approaches typically use encoder-decoder architectures to directly transform images across modalities, or rely on joint learning with shared representations [58,59]. However, these methods heavily depend on the availability of strictly paired data [60,61,62]. Contrastive learning techniques, such as SimCLR, CLIP, and MoCo, have proven effective for cross-modal representation learning [63,64,65]. Nonetheless, standard implementations of SimCLR require large batch sizes (ranging from 512 to 4096 examples), leading to significant computational demands [65,66,67]. Although momentum-based methods that use negative queue buffers can partially mitigate this issue, the challenge of adapting these approaches to medical data with limited paired data remains unresolved [68,69].

Many hybrid designs encounter practical challenges that are crucial for their use in ophthalmic applications. These challenges include increased architectural complexity, greater computational and memory requirements, sensitivity to limited labeled data in specialized patient groups, and the necessity for thorough validation of calibration and decision reliability, especially in borderline cases and when dealing with comorbid conditions [44,45,46].

Thus, there are no systems capable of: correctly modeling disease comorbidity through multi-label staging with suitable regularizations; performing consistent staging of DR using OCT data when fundus images are lacking; and maintaining high performance in the face of domain shifts and significant class imbalances that are typical of real clinical data.

The primary technical challenge addressed in this study is to achieve clinically consistent and calibrated diagnostics for retinal diseases using OCT. This challenge arises from three key factors: (i) the occurrence of multiple diseases simultaneously (multi-label comorbidity), (ii) the reliance on fundus photography as the clinically accepted grading standard for DR, and (iii) the limited availability of strictly paired OCT and fundus datasets in real-world clinical workflows.

Methodologically, this involves learning transferable representations across modalities without paired supervision, while also remaining robust to class imbalance and domain shifts caused by different scanners.

### Primary Clinical Promise and Contributions

To overcome these limitations, the main clinical promise of this work is to enable comprehensive, multi-label staging of retinal comorbidities—such as DR, DME, and AMD—using exclusively OCT imaging. It is achieved by bridging the diagnostic gap between OCT and fundus modalities with very few paired training data. Unlike traditional methods that treat these tasks separately, our solution integrates them into a unified framework. Specifically, we propose:

1. A hierarchical deep learning architecture that models the clinical dependency between retinal diseases (e.g., DR as a risk factor for AMD).
2. A cross-modal translation mechanism enables the model to learn specific features of fundus images, which are essential for DR staging, even when paired datasets are limited.
3. A contrast-equalization bridge that aligns domain-specific features, improving calibration and accuracy in real-world clinical settings.

## 2. Methods

The study implements a hierarchical modular system (HMS). After analyzing clinical needs and the limitations of existing approaches, we propose the following testable hypotheses:

**Hypothesis 1.** 
*A hierarchical architecture with specialized child staging models will enhance accuracy in classifying AMD and DR stages by decomposition of a complex task into more manageable subtasks.*

**Hypothesis 2.** 
*A loss function that employs class-balanced weighting is more effective than the standard Binary Cross-Entropy (BCE) for a multi-label task with class imbalance in OCT images.*

**Hypothesis 3.** 
*Utilizing a contrastive loss function enables training a cross-modal OCT-to-fundus image bridge even when strictly paired data is limited. Additionally, it demonstrates superior performance compared to alternative loss functions in terms of cross-modal alignment quality.*

**Hypothesis 4.** 
*Calibrating decision thresholds using the F1-optimization method across classes will reduce the expected model calibration error and enhance clinical applicability compared to using a fixed threshold of 0.5.*

**Hypothesis 5.** 
*The HMS system exhibits high stability across different scanners.*

### 2.1. Overview of the System Architecture

The HMS system has a three-level hierarchical architecture that breaks down complex diagnostic tasks into specialized subtasks and integrates their results through cross-modal representation alignment [70,71].

The problem is formulated as a multi-label classification task: for each class, an independent probability is predicted using a sigmoid model and binary cross-entropy. This method allows for the simultaneous representation of multiple diseases [72,73,74].

After establishing a general diagnosis, the algorithm moves through a series of detailed decision-making stages for the identified diseases. The basic (parent) multi-objective model is responsible for the initial classification of pathologies as either absent or present. After this, specialized components take over to perform specific tasks within a more focused context, based on the outputs from the parent model. This approach enhances the system’s interpretability, aids in debugging, and improves its tolerance for errors [75,76].

OCT is widely regarded as the “gold standard” for diagnosing AMD and plays a crucial role in quantitatively assessing retinal thickness and DME [77,78]. Hence, for staging AMD, this study employs the AREDS. In contrast, fundus photography is the most commonly used imaging technique for staging DR, as the ETDRS and ICDR scales were specifically developed for this type of imaging [79,80]. Following this, the architecture includes a fundus model for classifying DR stages. It features a trainable latent “bridge” that translates OCT features into the fundus encoder’s latent space.

Consequently, the structure of the HMS system consists of four key components: a parent model for the multi-target classification of OCT scans, an AMD model for staging, a fundus classification model that determines the stages of DR based on fundus images, and a cross-modal OCT-to-fundus bridge [65].

To ensure proper functioning of the latent bridge, a contrastive alignment of the OCT and fundus latent spaces is performed in advance. This process maximizes the similarity of positive pairs while dispersing negative pairs, thereby reducing the “modal gap” between the encoders. Contrastive learning formalizes this objective using the InfoNCE loss function. Simultaneously, the moment encoder and negative queue (MoCo) variants build a comprehensive and consistent dynamic dictionary of negative examples [64,65].

By using alignment and a trained bridge, the system can perform DR staging even with minimal fundus images. It achieves this by projecting the OCT embedding into the fundus space and then applying the fundus classifier head, ensuring the results align with the gold standard.

**Figure 1:**
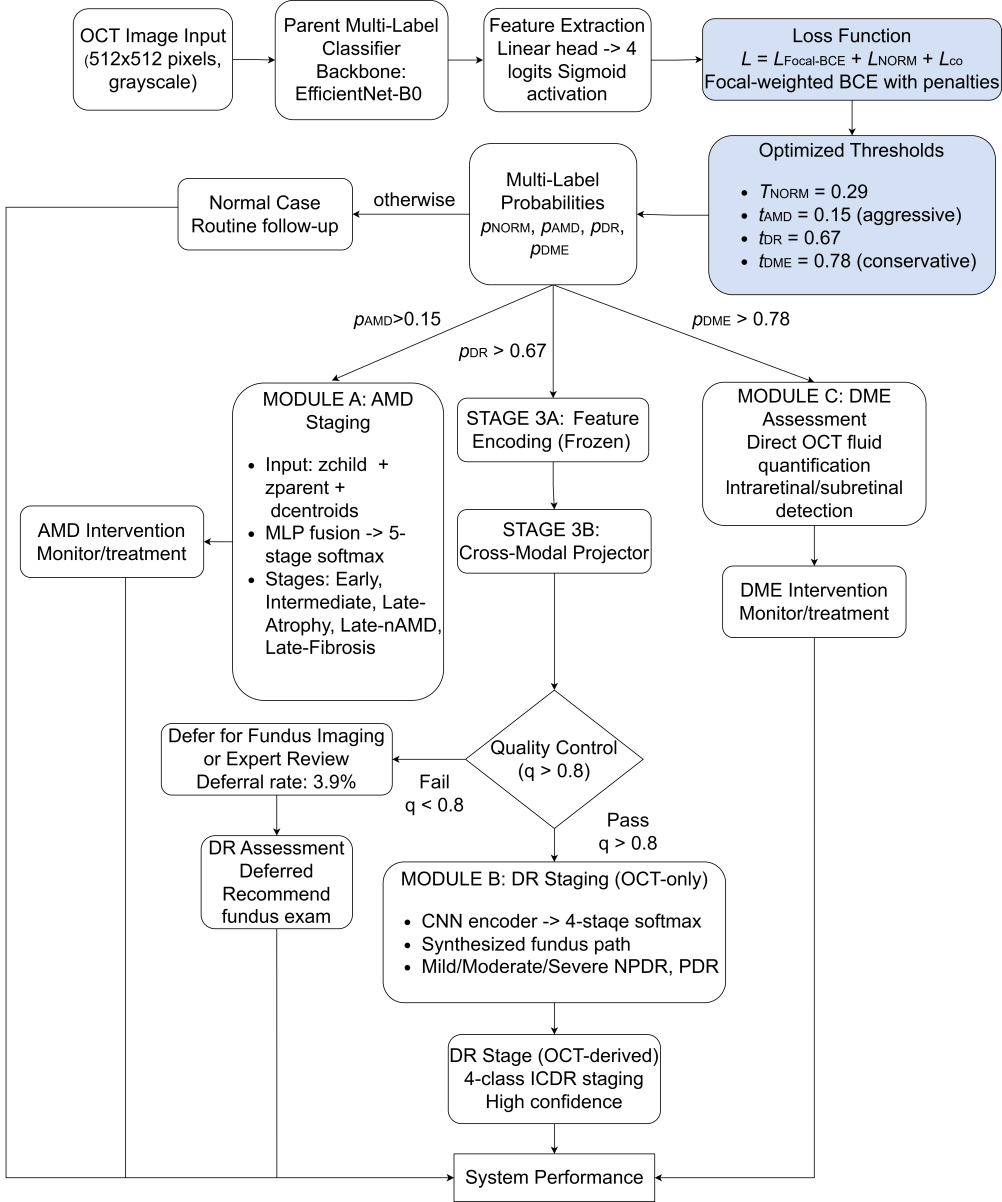
Architecture and hybrid workflow of the HMS system. White blocks represent the online inference pipeline, which includes modules activated during patient diagnosis. Shaded blocks denote offline optimization components, including loss functions and calibrated thresholds determined during training. Here, $p_{\mathrm{AMD}}$, $p_{\mathrm{DR}}$, $p_{\mathrm{DME}}$, are independent probabilities of diseases under consideration, and *q* is the cross-modal bridge projection (*q*).

The system architecture is illustrated in Figure 1. The diagram distinguishes between the active diagnostic path and the optimization components. The white blocks represent the inference pipeline: an input OCT scan is processed by the parent backbone to generate multi-label probabilities. Based on these probabilities, the sample is dynamically routed to one of the specialized staging modules (A, B, or C).

Shaded blocks denote the offline training and calibration components. The ‘Loss Function’ block illustrates the composite objective ($L_{Focal}+L_{Reg}$) used to train the backbone model. In contrast, the ‘Optimized Thresholds’ block contains the class-specific cutoffs (*T*) that are derived from the validation set through F1-score maximization. During the deployment phase, these thresholds serve as fixed gatekeepers for the routing logic.

**Figure 2:**
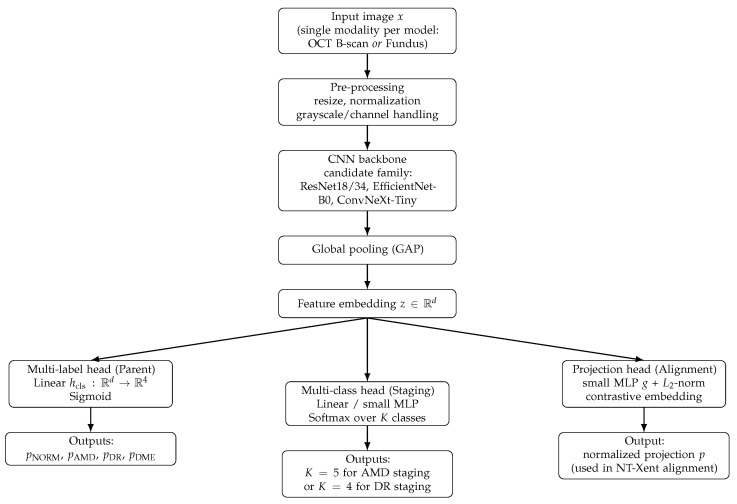
Schematic representation of the adopted CNN-based architectures used across HMS modules. A shared CNN encoder (pre-processing → backbone from the same candidate family → GAP) produces an embedding *z*. Different HMS components attach lightweight task-specific heads: (i) a multi-label sigmoid head for parent screening (4 outputs: NORM/AMD/DR/DME), (ii) a multi-class softmax head for staging (AMD: 5 classes, DR: 4 classes), and (iii) a projection head used during contrastive OCT–fundus representation alignment.

Across HMS components shown in Figure 2, we adopt a common CNN encoder template: input pre-processing, a backbone chosen from a controlled family (ResNet18/34, EfficientNet-B0, ConvNeXt-Tiny), and global average pooling to obtain an embedding *z*.

Task differences are introduced through lightweight heads attached to *z*: the parent model uses a 4-output multi-label sigmoid head (NORM/AMD/DR/DME), whereas staging models use a softmax head over *K* classes (AMD: K=5, DR: K=4).

During cross-modal training, an additional projection head (small MLP with normalization) is used to produce contrastive embeddings for OCT–fundus representation alignment; this head is an architectural add-on to the same CNN encoder.

To enhance transparency in our stage-wise error analysis beyond aggregate metrics, we provide confusion matrices for AMD (5-stage OCT-based staging) and DR (4-stage fundus-based staging) in the Supplementary Materials (see Section S4.12). In AMD, the primary source of confusion arises between the Early and Intermediate stages, with 3 out of 32 Early cases incorrectly predicted as Intermediate. It aligns with the borderline criteria outlined in the AREDS study. In DR staging, most misclassifications occur at stage boundaries. Notably, 5 of 45 PDR were misclassified as Moderate/Severe NPDR, indicating subtle visual signs of proliferation.

### 2.2. Operation of HMS Components

For multi-label or multi-objective classification, BCE with focal boosting is used. In the multi-objective setting, each label is treated as an independent binary problem with logits and a sigmoid activation function [81].

The focal loss function is designed to downweight well-classified examples while emphasizing more complex and rare cases. It achieves this by incorporating a modifying factor ${\left(1-p_{t}\right)}^{\gamma }$, which shifts the focus of learning towards the less frequent instances in the distribution. This approach enhances the model’s robustness to class imbalance [82]. In practical situations, imbalance can also be addressed by using class weights $\alpha_{c}$ [83].

For a batch of *N* examples and C=4 labels {No diseases (NORM), AMD, DR, DME}, with logits $s_{n,c}$ and probabilities $p_{n,c}=\sigma \left(s_{n,c}\right)$, where σ(·) is the sigmoid function, the focal-weighted BCE is expressed as:

$$L_{\text{Focal-BCE}}=\frac{1}{NC}\sum_{n=1}^{N}\sum_{c=1}^{C}\alpha_{c}{\left(1-p_{t,nc}\right)}^{\gamma }\left(-\log p_{t,nc}\right),\;p_{t,nc}=\left\{\begin{array}{cc} p_{n,c}, & y_{n,c}=1,\\ 1-p_{n,c}, & y_{n,c}=0, \end{array}\right.$$

which is the standard form for multi-objective problems and naturally generalizes BCE to the case with a focusing factor γ [82].

A comprehensive study was conducted to determine the most effective backbone architecture that involved ResNet18, ResNet34, EfficientNet-B0 and ConvNeXt-Tiny. The findings are detailed in Section 3. All architectures were pretrained on ImageNet, facilitating the transfer of essential low- and mid-level features and thus improving the convergence on medical images [84]. Additionally, the pretrained weights for grayscale input were obtained by averaging the weights of the first convolutional layer across the RGB channels.

#### 2.2.1. The Parent Model

The parent model is designed as a multi-label classifier. In this approach, the softmax function is replaced with independent sigmoid activations, using binary cross-entropy for each label. The sigmoid function, defined as $\sigma \left(x\right)=1/\left(1+e^{-x}\right)$, converts the logit (the raw output of the neural network) into a probability ranging from 0 to 1 for each class independently. This method effectively models disease comorbidity while avoiding the artificial exclusivity characteristic of softmax approaches [70,71,72,73,74].

The penalty, $R_{co}=E\left[p_{\mathrm{AMD}}\cdot p_{\mathrm{DR}}\right]$, is designed to discourage AMD–DR co-activation during training.

This measure was introduced as a heuristic specific to the dataset because there are few or no clear examples of OCT cases that are consistently co-labeled with both AMD and DR in the original sample, making the joint region difficult to identify.

It is important to note that $R_{co}$ is a *soft* regularizer and does not imply that AMD and DR cannot co-exist; in reality, comorbidity is clinically possible. To assess its practical impact, we conducted an audit of AMD–DR co-activation on held-out data (see Supplementary Materials Section S4.12). The results indicate that simultaneous high-confidence predictions are still achievable when there is strong evidence for both conditions.

This setup facilitates the joint modeling of multiple states while implementing clinical-logical regularizations that ensure consistency between predictions of “normal” and “pathological” conditions. To leverage the benefits of binary cross-entropy for multi-objective problems, we use Focal-BCE with label smoothing. This approach includes a focusing factor that enhances the contribution of “difficult” examples, addressing issues related to class imbalance.

To enhance clinical reliability, decision threshold calibration was performed using per-class F1-optimization [85]. This method produced a set of asymmetric thresholds that achieved an optimal balance between precision and recall for each diagnosis.

The detailed operating principle, routing logic to specialized modules, and interpretability requirements are outlined in the Supplementary Materials (Section S4.1). Class metrics, confidence intervals, and the evaluation protocol are presented in Table S5.

#### 2.2.2. The Child Model

To refine the diagnosis of AMD into five stages, a hybrid feature fusion approach is employed. This method combines local features extracted from a CNN with global context from the parent model and the prototypical geometry of the latent space. This approach aligns with multi-modal and multi-source feature-fusion practices, which consistently enhance quality by effectively integrating diverse features [86].

The central innovation involves transferring knowledge of DR stages from fundus images to OCT. Initially, two feature-extraction models are trained separately: the parent model and the Fundus model. The Fundus model classifies DR stages according to international standards into four categories: MILD_NPDR, MODERATE_NPDR, SEVERE_NPDR, and PDR. Its architecture employs a convolutional encoder similar to that of the parent model but is specially adapted for grayscale images.

The cross-modal bridge connecting OCT to Fundus is trained in two stages. First, a contrastive alignment of the latent spaces is performed to reduce the disparity between the two modalities. Next, a small regression projector is used to ensure a consistent mapping into the Fundus space. This modular approach improves the solution’s clinical explainability, manageability, and scalability, enabling independent enhancements to individual components without affecting the overall architecture.

The detailed operating principles of the child modules and the bridge are outlined in the Supplementary Material (see Section S4.2). Additionally, Tables S6 and S7 provide analyses of staging metrics, common errors, and the effects of prototypical regularization.

To ensure the reliability of the reported metrics, we followed a strict evaluation protocol. All performance metrics are reported as the mean ± standard deviation across five independent cross-validation folds. We also computed 95% confidence intervals (CIs) using non-parametric bootstrapping with 1000 iterations on the test set. To validate the performance gains of the proposed HMS architecture compared to the baselines (e.g., EfficientNet-B0), we conducted paired *t*-tests across folds. Differences were considered statistically significant at p<0.05. This approach confirms that the reported improvements in calibration (expected calibration error, ECE) and macro-AUROC are not due to random initialization or data splitting.

## 3. Results

This chapter describes the experimental program created to evaluate the HMS system. It includes developing a multimodal dataset that simulates clinical conditions, comparing baseline convolutional architectures, threshold calibration, and assessing both the parent model and specialized staging modules. Furthermore, the chapter features a cross-modal bridge analysis that examines probability calibration, computational efficiency, and verification across different scanners.

### 3.1. Creating a Data Set

The experimental dataset is a comprehensive multimodal collection of medical images comprising 8159 images across two main ophthalmic imaging modalities. The dataset reflects clinical practice, featuring 4047 OCT images with detailed multi-label annotations and 4112 fundus images. A notable aspect of this dataset is the limited yet important component of paired OCT and fundus images, with only 128 pairs, which represent 3.1% of the total dataset.

**Figure 3:**
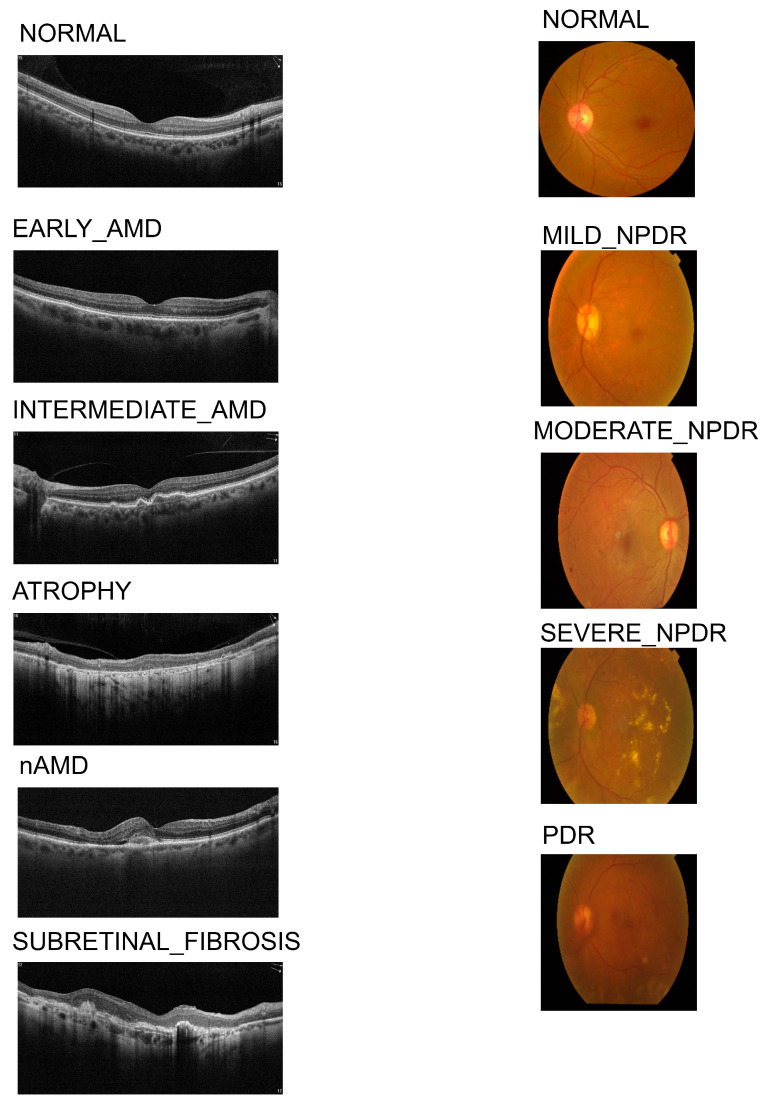
Representative examples of images analyzed in this study. The figure combines OCT B-scans illustrating AMD stages (Normal, Early AMD, Intermediate AMD, Atrophy, nAMD, Subretinal Fibrosis) and fundus images illustrating DR severity levels used for staging (Normal, Mild/Moderate/Severe NPDR, and PDR). All examples are anonymized and shown for illustrative purposes.

Figure 3 shows representative samples from both imaging modalities used in this work. The left column illustrates OCT B-scans corresponding to the AMD staging classes (Normal, Early AMD, Intermediate AMD, Atrophy, nAMD, and Subretinal Fibrosis), highlighting the characteristic structural changes observed in the macular region. The right column presents color fundus images corresponding to the DR staging scale (Normal, Mild/Moderate/Severe NPDR, and PDR), which is treated as the reference grading scheme for DR severity in our experiments.

To maintain the integrity of the “unpaired” learning paradigm, we strictly separated the 128 paired OCT–fundus samples from the training process. These pairs were used exclusively for testing and validation purposes. The cross-modal bridge and all encoders were trained using unconnected sets of OCT and fundus images, relying solely on contrastive alignment and cycle-consistency losses without any ground-truth pairing. The paired samples served as a hold-out test set to objectively assess the quality of the latent feature translation. They were used to calculate alignment metrics (e.g., feature cosine similarity) and to select the best model checkpoint, but not to optimize network weights directly. This protocol ensures that the reported performance accurately reflects the system’s ability to generalize in a truly unpaired clinical setting.

To ensure comprehensive coverage of various pathological conditions, we compiled the dataset from three distinct sources. The first source is an in-house clinical dataset obtained from the Optimed Laser Vision Restoration Center in Ufa, Russia, consisting primarily of the OCT images, totaling 2185. The second source is the publicly available Optical Coherence Tomography Image Database (OCTID), developed by the University of Waterloo [87]. The third source is the OCT-AND-EYE-FUNDUS-DATASET, which was created explicitly for the study of DME and DR. This collection includes 1548 fundus images and 1113 macular OCT images [88].

### 3.2. Label Harmonization and Grading Protocol

To integrate three data sources (in-house OCT, OCTID, and OCT–fundus data), we defined a unified label space that aligns with the tasks discussed in this paper. It consists of two main components: (i) multi-label parent prediction for OCT, which includes four binary labels {NORM, AMD, DR, DME}, and (ii) fundus-based DR staging, which categorizes into four stages {Mild NPDR, Moderate NPDR, Severe NPDR, PDR}. For transparency, Table 1 summarizes how native labels were mapped.

#### Harmonization Rules

- *In-house OCT:* AMD staging labels are based on AREDS-derived OCT interpretation; these stages correspond to the binary AMD label used for the parent model, whereas NORM/DR/DME labels adhere to the internal clinical annotation protocol established during dataset curation.
- *OCTID:* the original dataset provides disease-level OCT categories, distinguishing between options such as Normal and DR. As a result, OCTID samples were used only to support the corresponding binary parent labels (NORM or DR) and were not used for DR staging.
- *OCT–fundus dataset:* fundus images are utilized for DR staging based on the ICDR scale. In contrast, OCT images are used to establish the parent labels (NORM, AMD, DR, or DME) according to the diagnostic framework provided by the dataset.

The final dataset, including class distribution across modalities, imbalance metrics, source descriptions, and a five-fold cross-validation strategy among patients, is presented in the Supplementary Materials (Section S4.3, Tables S1 and S2).

A critical methodological feature is the strict separation of patient identifiers, which prevents information leakage between the training and test sets and ensures a fair assessment of the system’s ability to generalize to new patient data.

### 3.3. A Comparison of Backbone Architectures for Choosing a Base Classifier Model

To systematically evaluate and justify the selection of a backbone convolutional architecture, a thorough comparative study was conducted on four modern architectures: ResNet18, ResNet34, EfficientNet-B0, and ConvNeXt-Tiny. These architectures were chosen for their widespread use in medical imaging and for the balance they provide among accuracy, computational efficiency, and the number of trainable parameters [48,89].

To ensure methodological rigor, all models were trained using a consistent experimental protocol. The same optimization hyperparameters and data augmentation strategies were applied uniformly across all models. The optimizer used was AdamW, configured with a learning rate of $3\times 10^{-4}$ and a cosine annealing scheduler [90,91].

The mini-batch size consisted of 64 images. To enhance the model’s robustness, augmentation techniques were applied, including random horizontal flips, $\pm 10^{\circ }$ rotations, and adjustments to brightness and contrast within ±0.1 [92,93,94].

For comparison purposes, we utilized the unified macro-F1 and micro-F1 metrics, which are standard aggregates in multi-class classification. These metrics are derived from micro- and macro-averaged Precision, Recall, and F1 scores across different classes [95]. Additionally, we employed multi-class ROC-AUC with binarization (where *p* > 0.5) [96]. The dataset was split into training, validation, and test sets at 80/10/10. We chose BCEWithLogitsLoss (binary cross-entropy with logits) as our loss function [97,98]. Detailed information, including Macro/Micro-F1 metrics, Hamming loss, Jaccard index, and metrics for computational efficiency, is available in the Supplementary Materials (Section S4.4, Table S3).

The EfficientNet family of architectures, which are designed for compactness and efficient scaling, outperformed deep residual networks in terms of classification performance while using significantly fewer parameters. In clinical applications, it is essential to balance classification accuracy with computational demands, as the chosen architecture influences inference latency. A key methodological finding is that the benefits of compact architectures remained consistent across both the validation set and cross-validation, demonstrating their stability in generalization. The selected backbone architecture provided the foundation for developing all subsequent components of the hierarchical system.

### 3.4. Calibrating Thresholds to Compensate for Class Imbalance

To address class imbalance in a multi-label classification setting, it is crucial to optimize class-specific thresholds [99]. The standard threshold of 0.5 is often insufficient, especially in situations with significant class imbalance. To overcome this challenge, we conducted a systematic comparison of four methods for adaptive threshold calibration. For transparency, we present the metrics of the parent model, evaluated with both the fixed 0.5 threshold and class-specific threshold calibration (Table S4).

To ensure that our high macro-F1 score of 0.989 reflects genuine probability calibration rather than threshold artifacts, we independently evaluated calibration curves and the expected calibration error (ECE) on the held-out test fold. The ECE, which stands at 2.1 ± 0.4, is significantly below the 5% threshold commonly accepted in medical literature as an indicator of good calibration [100]. Calibration remains consistent across all ranges of predicted probabilities (Table S4 in Section S4.5 and Figure S2 in the Supplementary Materials), confirming that the reliability of decisions is stable for both high-confidence and borderline cases. Additionally, a comparison of alternative calibration methods, such as temperature scaling and isotonic regression, shows that optimizing per-class F1 thresholds provides superior performance while either maintaining or improving probability quality.

All thresholds were tuned exclusively on the validation split (validation fold in cross-validation) and were never optimized on the held-out test split/fold. Test metrics were computed by applying the thresholds obtained from the corresponding validation split/fold.

The F1-optimization methodology, applied independently to each class, demonstrated superior performance to other approaches. It effectively achieves an optimal balance between sensitivity and specificity across different disease prevalences. This strategy employs an aggressive detection threshold of 0.15 for AMD to minimize the risk of missing cases in the late stages of the disease, when the possibility of vision loss is exceptionally high. In contrast, a more conservative threshold of 0.78 is applied for DME to prevent unnecessary interventions in patients with significant comorbidities [101]. The optimal thresholds established are as follows: NORM = 0.29, AMD = 0.15, DR (diabetic retinopathy) = 0.67, and DME = 0.78. These thresholds account for the class imbalance in the dataset, where AMD represents over 50% of the OCT samples, while DME accounts for only 8.6% of the cases.

Methodologically, this approach employs the principle of cost-sensitive learning. Implementing individually calibrated thresholds led to significant performance improvements compared to the baseline threshold of 0.5.

### 3.5. Outcomes of the Parent Model Operation

The multi-label classification model for parents showed strong performance on the stratified test set, successfully identifying multiple comorbid conditions in a single diagnostic cycle. Comprehensive metrics are available in the Supplementary Materials, particularly in Section S4.6, Table S5.

Because AMD–DR co-labeled OCT cases are scarce in our dataset, we additionally report an AMD–DR co-activation audit to ensure that the model is not compelled to adhere to strict mutual exclusivity (see S12. Section S4.12 in Supplementary Materials).

On the internal test set, the AMD class achieved near-ceiling performance, with precision and recall metrics of 1.000 (95% CI: 1.000–1.000), likely due to the high prevalence of distinct morphological features in the clinical cohort. This high performance is likely due to the clear and distinct morphological features prevalent in the clinical cohort. The Normal class (NORM) demonstrated a recall of 1.00 and a precision of 0.993, indicating only one false-positive result out of 136 cases. This result aligns with a conservative screening strategy focused on minimizing the risk of overdiagnosis. The DME class recorded a recall of 0.978, resulting in one missed case out of 50. It may be attributed to borderline cases that involved minimal intraretinal fluid and were close to the clinical criteria for DME with central involvement [102]. In contrast, the DR class achieved a recall of 0.990, with two false-negative results, both linked to MILD_NPDR and presenting with just one microaneurysm. It reflects the limited informational value of OCT in staging DR [85,103].

The performance distribution emphasizes the importance of adaptive calibration tailored to each specific diagnosis.

### 3.6. Results from the Specialized AMD Staging Module

The specialized module developed for diagnosing AMD showed impressive diagnostic performance, achieving an overall accuracy of 98.3±1.4%. Detailed results can be found in the Supplementary Materials, specifically in Section S4.7, Table S6. A significant finding is that late-stage AMD, which is characterized by clear morphological changes such as atrophy, subretinal neovascularization, and fibrosis, is classified with nearly perfect accuracy. However, distinguishing between the early and intermediate stages poses a systematic challenge due to the continuous nature of disease progression and the subjective boundaries between these stages, even among experts. This difficulty underscores the inherent uncertainty of the AREDS clinical classification scheme.

A key finding of this study is that the model demonstrates high precision at all stages, which is essential for minimizing false-positive diagnoses of late-stage conditions that necessitate aggressive treatment. The model’s performance is comparable to previously published results for five-class AMD staging, while also providing the unique advantage of unified multi-label diagnostics and improved probability calibration.

### 3.7. The Results from the Specialized Module for Staging DR

The developed model for classifying DR stages using fundus images achieved an overall accuracy of 94.8±0.9%. A significant methodological advancement is that this model can stage DR according to the international ICDR classification based solely on synthesized fundus representations derived from OCT scans. It demonstrates a successful cross-modal transfer of diagnostically significant features.

The performance distribution by stage shows a clear pattern: the intermediate stages of nonproliferative retinopathy can be classified with high accuracy, driven by distinct morphological features such as microaneurysms and hemorrhages. However, borderline cases between stages need expert verification, as they depend on subjective clinical criteria. Notably, the performance achieved is comparable to that of models trained directly on real fundus images. For detailed metrics, please refer to the Supplementary Materials, specifically Section S4.8, Table S7.

### 3.8. The Cross-Modal Bridge and Analysis of Cross-Modal Inconsistencies

The contrastive alignment of OCT and fundus images was achieved using the NT-Xent (Normalized Temperature-scaled Cross-Entropy) loss function, along with a pulse encoder set to m = 0.999 and a negative queue containing 512 samples [65]. The training process exhibited a two-phase dynamic typical of contrastive learning. In the first 20 epochs, the loss function decreased rapidly on the training set. Following this period, the Recall@1 metric, which measures the accuracy of retrieving the first nearest neighbor, gradually improved, reaching its highest value at the 54th epoch. A peak Recall@1 value of 0.411 indicates a successful alignment of the latent-space geometries for the two modalities, thereby facilitating reliable cross-modal image retrieval [104].

The cross-modal bridge between OCT and fundus images was trained using a multi-component loss function that combined seven regularization components: mean squared error (MSE), cosine closeness (Cosine), Kullback-Leibler divergence (KL-divergence), InfoNCE contrastive loss, prototype loss (Prototype), maximum mean discrepancy (MMD), and correlation alignment (CORAL) [105,106,107]. The training process is described in detail in the Supplementary Material, specifically in Section S4.9.

The cross-modal bridge is trained using a composite objective function that aligns representations at three distinct levels. To prevent an arbitrary combination of terms, we organize the loss into three functional groups:

- Geometric Alignment ($L_{MSE},L_{Cosine}$): Directly minimizes the metric distance and maximizes angular similarity between projected OCT and fundus embeddings.
- Contrastive & Structural Constraints ($L_{InfoNCE},L_{Proto}$): $L_{InfoNCE}$ ensures discriminative separation of positive/negative pairs, while $L_{Proto}$ clusters embeddings around class centroids to preserve semantic separability.
- Statistical Distribution Matching ($L_{KL},L_{MMD},L_{CORAL}$): These regularizers align the higher-order moments of feature distributions to help reduce domain shift.

The final objective is a weighted sum: $L_{total}=\lambda_{1}L_{MSE}+\lambda_{2}L_{Cos}+\ldots$. We determined the optimal configuration ($\lambda_{InfoNCE}=1.0$, $\lambda_{MSE}=10.0$, others ∈[0.1,1.0]) through grid search on the validation set. Exact weights and implementation details are included in the Supplementary Materials, Section S4.9.

To address the concerns about the complexity of the bridge objective, we conducted a systematic leave-one-out ablation using identical training settings (see Supplementary Materials, Table S11). The results demonstrate a clear hierarchy of the importance of each component:

- Critical components: Removing $L_{InfoNCE}$ or $L_{Cosine}$ results in the largest drops in fundus agreement (3.91 and 3.13 percentage points, respectively). This confirms that contrastive pressure and angular alignment are the primary contributors to cross-modal transfer.
- Secondary regularizers: Removing $L_{Proto}$, $L_{MSE}$, or $L_{MMD}$ leads to minor but significant decreases in agreement (2.35, 1.57, and 1.56 percentage points, respectively), suggesting that these terms help stabilize the mapping and enhance semantic consistency.
- Lightweight distribution matching: Removing $L_{KL}$ has a relatively modest effect of 0.78 percentage points, indicating that it primarily functions as an auxiliary regularizer in our context.

Overall, the data in Table S11 (Supplementary Materials) demonstrate that two terms dominate cross-modal alignment, while the remaining terms provide consistent secondary gains.

There was a significant improvement in the fundus consistency score, which increased from 0.815 to 0.984 between epochs 18 and 30. This change indicates a qualitative enhancement in the bridge’s ability to produce semantically consistent representations. The results demonstrate that the multi-component learning strategy not only facilitates geometric alignment of feature spaces but also achieves a high level of semantic correspondence between OCT and fundus imaging modalities. This correspondence is crucial for subsequent cross-modal diagnostic tasks related to retinal diseases [108].

An ablative study emphasizes the significance of contrastive alignment. The key methodological finding is that information-theoretic components, such as InfoNCE and KL-divergence, greatly enhance the quality of representation alignment. In contrast, geometric components such as MSE and Cosine play an important but less prominent role in maintaining metric consistency. Removing any of these components results in a statistically significant decline in performance, highlighting the synergistic effect of optimizing multiple components and the necessity of balancing the different aspects of representation alignment.

To assess the effectiveness of cross-modality transfer between OCT and fundus images, it is crucial to identify cases where the bridge model struggles to accurately project OCT data into the fundus representation space. Key challenges include artifacts that affect image quality, physiological variations at the boundaries of clinical staging criteria, and the anatomical limitations of each modality’s field of view. It is important to recognize that these discrepancies do not indicate failures of the algorithm; instead, they highlight a fundamental incompleteness in the information provided by one modality compared to the other.

Quantitative analysis reveals that fewer than 4% of paired data cases exhibit significant discrepancies, demonstrating the method’s overall stability. However, the identified issues point to several critical clinical situations that require further expert verification. Additional information about this research step can be found in the Supplementary Materials, specifically in Section S4.9, Table S13, and Figure S1.

#### Failure Taxonomy for OCT-Only DR Staging When Fundus Is Absent

While the proposed OCT-to-fundus latent bridge enables ICDR-consistent DR staging in most cases, it is important to note that OCT-only staging does not always equate to fundus-based grading in all clinical situations. Specifically, some critical signs identified by the ICDR are either located outside the typical macular OCT field of view or are presented mainly as en-face microvascular patterns, which standard structural OCT B-scans may not reliably capture [21,109].

We provide a clear failure taxonomy that identifies situations in which fundus grading is accurate, but OCT-only staging may be incorrect. Each category is linked to underlying limitations in retinal coverage (field of view) and/or the visibility specific to each modality.

Table 2 presents a qualitative failure taxonomy based on ICDR fundus-based criteria alongside our paired hold-out discrepancy review (see Supplementary Materials, Table S13). It highlights instances where a limited macular OCT field-of-view may contribute to disagreements between fundus and OCT findings [21,109].

These categories align with our cross-modal discrepancy analysis of the paired hold-out set (see Supplementary Materials Section S4.9, Table S13). It includes representative cases where disagreements can be reasonably attributed to limited retinal coverage or artifacts.

### 3.9. Calibration Assessment, Risk Interpretation in Clinical Scenarios, and Computational Efficiency

The calibration evaluation shows high reliability for probabilistic predictions. The Expected Calibration Error (ECE) on the held-out test set is 2.1±0.4%, which is significantly below the 5% threshold commonly accepted in medical literature [100]. To ensure that the high Macro-F1 score of 0.989 is not solely due to threshold tuning, we conducted a comparative analysis of calibration methods on the held-out test set. For further details, please refer to the Supplementary Materials, specifically Tables S4 and S15.

As shown in Table S15 (in Supplementary Materials), the proposed per-class F1-threshold tuning outperforms standard post-hoc calibration methods. Specifically, it achieves a lower ECE (2.1%) compared to Temperature Scaling (2.4%) and Isotonic Regression (2.2%). Additionally, it maintains superior discrimination with a Macro-F1 score of 0.989, surpassing the scores of 0.968 and 0.976 for Temperature Scaling and Isotonic Regression, respectively. It indicates that the model’s predicted probabilities are well-calibrated and that the decision thresholds selected on the validation set generalize effectively to unseen test data without overfitting.

Experiments were conducted to evaluate computational efficiency using a hardware configuration that includes an NVIDIA RTX 3060 GPU and a Ryzen 7 3700X CPU. This modular design allows tasks to be divided by specialization and executed either independently or in a pipeline. This approach enhances portability, interpretability, and scalability across various clinical scenarios. The complete pipeline processes an OCT volume comprising 128 B-scans in approximately 3.15 s on the GPU, achieving an average latency of 24.6 ms per image. This performance meets real-time visualization requirements without hindering clinical workflow throughput [110]. A detailed description of the tests can be found in the Supplementary Materials, specifically in S10. Section S4.10, Figure S2, and Table S16.

### 3.10. Comparative Analysis of Model Effectiveness in Diagnosis and Staging

**Figure 4:**
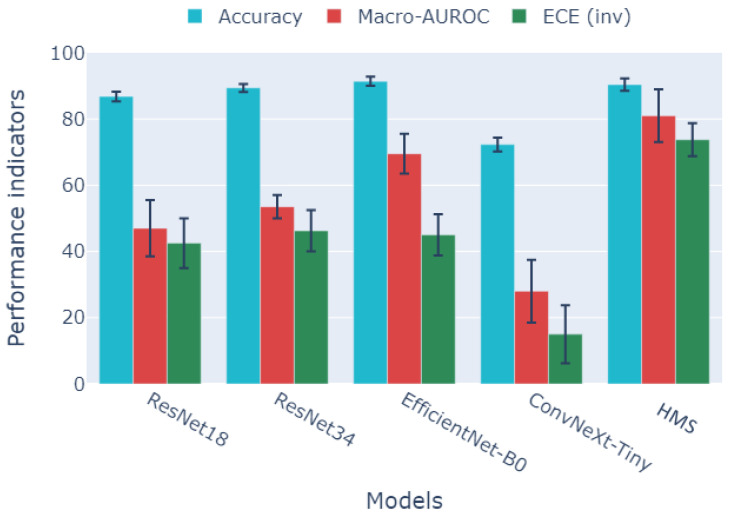
A comparison of the performance of the HMS system with contemporary baseline methods.

Figure 4 presents the comparison of the HMS system with state-of-the-art baselines. To address potential overconfidence in single-point estimates, we report all metrics with seed variance and 95% CIs (detailed in Supplementary Tables S3 and S13). While EfficientNet-B0 achieves a competitive peak accuracy, the proposed HMS model demonstrates a statistically significant improvement in probabilistic reliability. Specifically, HMS reduces the Expected Calibration Error (ECE) to 2.1±0.4% compared to 4.3–5.3% for standard CNNs (p<0.01, paired *t*-test), identifying a critical advantage for clinical decision support. In terms of discrimination, HMS achieves a macro-AUROC of 0.965 (95% CI: [0.958, 0.972]), which is superior to the monolithic baselines in multi-label separation tasks. This distinction emphasizes the difference between optimizing for accuracy and achieving high-quality probabilistic calibration [47].

### 3.11. Cross-Scan Validation and Robustness to Domain Shifts

To evaluate the generalizability of the HMS system across various hardware configurations, cross-scanner validation was performed using a clinical dataset from the Optimed Laser Vision Restoration Center in Ufa, Russia. This dataset consisted of 2185 OCT images. The cross-domain validation studies, summarized in the Supplementary Materials, specifically in Section S4.11, Table S17.

The results reveal an asymmetric pattern of cross-scan generalization. When transferring from the Avanti XR scanner to the REVO NX scanner, we observe a significant increase in accuracy to 86.1%, along with a discriminatory power (AUROC of 0.896). Conversely, transferring in the opposite direction, from REVO NX to Avanti XR, leads to a noticeable drop in performance, with accuracy falling to 74.7% and AUROC decreasing to 0.769. The HMS hierarchical model shows a systematic advantage over the leading baseline method, EfficientNet-B0, with improvements of 7.2 to 7.3 percentage points in both transfer directions. It highlights the hierarchical architecture’s enhanced robustness in handling domain-specific variations. These findings support existing evidence that multi-stage and adaptive approaches are effective for cross-domain generalization. While some performance degradation during transfers between different scanners is unavoidable due to variations in optical characteristics, resolution, noise patterns, and image acquisition parameters, the hierarchical approach significantly mitigates this degradation. It achieves this by enabling modular specialization and contrast alignment.

To enhance external validation beyond cross-scanner transfer, we conducted a Leave-One-Source-Out (LOSO) evaluation across the three OCT data sources used in this study: In-house, OCTID, and OCT–Fundus Dataset. In each LOSO setting, we trained the parent OCT multi-label classifier on two of the data sources and tested it on the third, held-out source. We used the same preprocessing pipeline and validation-only threshold-tuning protocol as in the primary experiments (see Supplementary Materials, S11. Section S4.11 and Table S18).

As expected, LOSO generalization testing is stricter than mixed-source cross-validation. We observed a decline in performance relative to the internal baseline because the held-out source introduces simultaneous changes in both acquisition characteristics and pathology composition. However, the results remain consistent across unseen sources, suggesting that the model does not depend exclusively on source-specific shortcuts. It supports the robustness claims we reported in the cross-scanner analysis.

## 4. Discussion

The proposed hierarchical structure may resemble a standard modular cascade, but it has distinct advantages over a monolithic multi-task backbone that shares features. Its novelty lies in three key mechanisms: (i) clinically motivated conditional routing (from parent diagnosis to specialist staging), which prevents negative transfer between unrelated tasks; (ii) geometry-aware feature fusion in child staging, where specialized features are enhanced with global parent context and prototype-based geometric constraints (refer to Supplementary Material, Section S4.2); and (iii) a cross-modal latent bridge that facilitates OCT-only DR staging by projecting OCT embeddings into the fundus space, ensuring alignment with the clinical gold standard.

The design choices made in this study have led to improved performance across several clinically relevant metrics. As illustrated in Figure 4, HMS exhibits competitive discrimination performance (macro-AUROC) when compared to robust single-backbone baselines, such as EfficientNet-B0. Additionally, HMS consistently produces better-calibrated results, as evidenced by lower ECE scores. These advantages also correlate with enhanced robustness to domain shifts, which is anticipated given the system’s modular specialization and explicit cross-modal alignment. Specifically, our cross-scanner evaluation (detailed in Supplementary Material, Table S17) shows that HMS maintains stable accuracy and discrimination when transferring between devices. In contrast, a monolithic baseline experiences a significant drop in performance, reinforcing the idea that the hierarchical structure helps minimize scanner-specific variability in the learned representation.

To align the baseline comparison directly with our core claims, we provide a concise summary of claim-matched competitive baselines in the Supplementary Materials (Table S12). Specifically, this table distinguishes a strong calibrated multi-label OCT baseline (focal-style multi-label learning with validation-only threshold tuning and ECE-based calibration assessment) from an unpaired cross-modal baseline (bridge variant without contrastive pressure), improving attribution of the reported gains.

These factors are crucial for ensuring the clinical reliability of decisions made under uncertainty (for detailed metrics and tables, refer to Sections S4.4–S4.6). This aligns with recent findings that attention-aware analysis is essential for practical interpretability checks in multi-modal systems [111], particularly when verifying logic in the presence of comorbidities.

A significant distinction between HMS and studies such as VisionTrack is its ability to perform DR staging using OCT rather than relying on fundus imaging. It is achieved through a two-stage cross-modality alignment process and a latent bridge, eliminating the impractical requirement for strictly paired multimodal data in clinical settings. It supports the validity of the first hypothesis outlined in Section 2.

Using class-balanced weights and focal BCE enhances sensitivity to rare pathologies in multi-label settings. This improvement is evidenced by higher overall metrics and increased robustness to class imbalance, as detailed in the Supplementary Materials, Sections S4.5 and S4.6. When combined with modular decomposition, these methods produce significantly better performance across various classes. This approach accounts for epidemiological factors and the expression of visual features, reflecting real clinical frequencies and helping reduce the omission of rare conditions. Furthermore, the representation geometry generated in child modules, along with prototypical regularization, enhances interclass separability and robustness against the “long tail” of distributions. This outcome confirms the validity of the second hypothesis presented in Section 2.

The third hypothesis suggests that a contrastive loss function can facilitate the training of a cross-modality bridge between OCT and fundus images without requiring strictly paired data. This idea is supported by evidence showing that DR can be staged by mapping it into a latent fundus space and classifying it according to the ICDR standards. For further details, please refer to the Supplementary Materials, specifically Section S4.9. The approach involves two stages: first, employing NT-Xent with a momentum encoder and a queue of negative samples; second, using a regression projector with a multi-component loss function. This framework ensures robust spatial alignment and effective transfer of important diagnostic features between the two modalities. To ensure clinical safety, a cosine similarity threshold of 0.8 has been established.

We further clarify the operational meaning of this QC rule and report a small sensitivity analysis around the chosen threshold (Supplementary Materials, Section S4.9, Table S14). Specifically, lowering the threshold decreases the manual workload but may overlook clinically significant cases of severe under-staging patterns (such as Severe NPDR/PDR). On the other hand, a higher prevalence of artifacts results in more cases being deferred for manual review, which is intentionally designed to ensure a conservative fail-safe approach.

As a result, 3.9% of cases are flagged for manual verification due to artifacts and borderline manifestations. This approach minimizes the risk of false positives while enabling automation in 96.1% of cases. Illustrations of these inconsistencies can be found in the Supplementary Materials, Section S4.9.

Class-specific F1 threshold calibration significantly reduces calibration error and enhances clinical applicability compared to a fixed threshold of 0.5. This improvement is evidenced by a higher macro-F1 score of 0.989, compared to 0.923 at the 0.5 threshold, and a lower ECE of 2.1±0.4%, in contrast to the typical 4–6% seen with uncalibrated CNNs. Calibration curves and summaries are available in Section S4.10 of the Supplementary Materials. Furthermore, the improved calibration remains effective across a wide range of probabilities, which enhances the interpretability of risk in both high-sensitivity and borderline cases. It is crucial for making clinical decisions under uncertainty. Overall, these findings confirm the validity of the cost-sensitive postprocessing approach for tasks characterized by pronounced class imbalance and unequal error costs, thereby supporting the fourth hypothesis outlined in Section 2.

The fifth hypothesis regarding the robustness of transfers between OCT scanners is partially confirmed. The system maintains a clinically acceptable level of accuracy during these transfers, although we observe asymmetric degradation. Specifically, the accuracy is 86.1% when transferring from Avanti XR to REVO NX and 74.7% when transferring from REVO NX to Avanti XR. Additionally, HMS consistently outperforms EfficientNet-B0 by 7.2 to 7.3 percentage points in both directions. This result underscores the advantages of hierarchy and prototypical regularization for cross-domain generalization (see Supplementary Materials, S11. Section S4.11 for full tables). The remaining decrease in within-domain performance can be attributed to differences in optical parameters, noise-reduction algorithms, and resolution between manufacturers (Optovue vs. Optopol), highlighting the need for further domain adaptation.

In contrast to systems that focus on single-modality scenarios and metadata integration, such as VisionTrack, HMS addresses broader cross-modality transfer without relying on paired data. It explicitly evaluates calibration and robustness to domain shifts, thereby enhancing clinical validity and the transferability of results. The presented integral indices (macro-F1 = 0.989±0.006; micro-F1 = 0.994±0.003; Jaccard index = 0.996±0.001) should be interpreted with caution due to differences in datasets and protocols. However, they confirm the competitiveness of the proposed architecture for multi-target OCT classification.

The system combines high accuracy, calibrated probabilities, and modular explainability to support a range of scenarios, including screening for primary AMD, DR, and DME. It also aids in AMD staging according to the AREDS guidelines, allows for cross-modality staging of DR without the need for fundus images, and enables DME monitoring through interpretable risk scores.

The confusion matrices indicate that the residual errors are not random; instead, they are structured around clinically recognized boundary cases. In AMD staging, the most significant confusion occurs between Early and Intermediate AMD. It is expected that the AREDS boundary relies on continuous morphological attributes, such as drusen size, which can be somewhat subjective when distinguishing between these stages near the threshold.

Crucially, the model does not confuse early AMD with late AMD phenotypes, suggesting that the learned representation preserves the ordinal nature of disease progression and focuses errors on adjacent, morphologically similar stages.

In DR staging, most errors occur during transitions between adjacent ICDR stages. The most significant recall loss is seen in the PDR stage. It aligns with the established challenge of detecting minor or peripheral neovascularization and borderline proliferative signs on standard color fundus images, particularly when the lesions are subtle.

Thus, the confusion structure supports a clinically realistic deployment approach: automated staging is most reliable for distinctly separated stages, whereas borderline transitions should be considered uncertain cases that may require expert verification.

The entire processing pipeline can analyze 128 B-scans in approximately 3.15 s on a GPU, yielding about 24.6 ms per image. Each module can operate independently or in tandem within the pipeline. The system delivers a combined performance of 7.72 GFLOPs and uses 37.0 million parameters, making it suitable for real-time applications. However, CPU performance may limit functionality in scenarios without specialized hardware (refer to Supplementary Materials, SM Section S4.10 for exact profiles). These performance characteristics make the system practical for integration into a clinical workflow, with the flexibility to adapt to different resource availability.

While the HMS system demonstrates robust performance, several limitations must be considered for clinical translation:

1. Anatomical Field-of-View (FOV) Constraints. The most significant clinical limitation is the discrepancy in FOV between macular OCT and wide-field fundus photography. As detailed in our failure taxonomy (Table 2), peripheral diabetic lesions (e.g., NVE elsewhere) are optically invisible to standard macular OCT. Consequently, our OCT-only DR staging is inherently limited to *macula-correlated* signs. To address this issue, we have implemented a QC gate (q<0.8) that typically flags such ambiguous cases for mandatory fundus review. However, there remains a risk of under-staging diseases that are present exclusively in the peripheral retina.
2. Unpaired Training Risks. Relying on unpaired cross-modal translation carries the risk of semantic misalignment, leading the system to generate plausible fundus features that do not actually exist in the OCT images. While our contrastive constraints and validation on the 128-pair holdout reduce this risk, the system’s reliability when encountering rare, anomalous pathologies that were not included in the training data remains untested.
3. Dataset and Labeling Bias. The study relies primarily on single-center annotations (Optimed), which may introduce institutional bias. Furthermore, the dataset-specific heuristic penalty $R_{co}$ was implemented to stabilize training due to the limited availability of AMD–DR co-labels. Although our audit (see Section S4.12) confirms that strong co-signals are still detected, we recommend relaxing this constraint as larger, more diverse comorbidity datasets become available.

Future steps will involve multi-domain contrastive learning with adversarial alignment, the accumulation of paired data addressing real-world AMD and DR comorbidity to eliminate the $R_{co}$ penalty. Additionally, we will implement uncertainty assessment methods, such as ensemble techniques, Monte Carlo dropout, and Bayesian approaches. The development of attention mechanisms will also be a priority, as it will enable visually explainable decisions. This program aims to enhance the system’s transferability, trustworthiness, and usability in multi-center and resource-constrained environments.

## 5. Practical Deployment Workflow and Audit Logic

To facilitate real-world clinical use, we provide an actionable workflow that specifies (i) when OCT-only decisions are acceptable, (ii) when fundus imaging is required, and (iii) which confidence/quality criteria trigger referral. Our system outputs calibrated multi-label probabilities for {NORM, AMD, DR, DME} and directs positive cases to specialized modules. The decision thresholds are determined solely from the validation fold via per-class F1 optimization, while the calibration quality is evaluated on a separate held-out test fold.

### 5.1. When OCT-Only Is Sufficient vs. When Fundus Is Required

**Step 1 (OCT acquisition and parent screening).** In an OCT scan, the parent model predicts probabilities for NORM, AMD, DR, and DME. If all pathology probabilities remain below their calibrated operating thresholds, the case is classified as low risk and can be scheduled for routine follow-up.
**Step 2 (Specialist staging).** If AMD is detected, the AMD specialist module performs OCT-based staging using AREDS-based interpretation. If DR is detected, the system attempts OCT-only DR staging via the OCT → Fundus latent bridge.
**Step 3 (Quality control gate for OCT-only DR staging).** OCT-only DR staging is accepted only if the cross-modal projection quality score (cosine similarity between the projected OCT embedding and the reference fundus embedding) satisfies q≥0.8, which covers 96.1% of paired hold-out cases in our study. If q<0.8, the system defers staging and recommends fundus imaging and/or expert review, acting as a conservative fail-safe.

### 5.2. Referral Triggers and Safety-Oriented Deferral Rules

In deployment, borderline or clinically high-impact scenarios should be handled conservatively:

- Fundus required: (i) q<0.8 for OCT-only DR staging; (ii) suspected severe DR/PDR near stage boundaries; (iii) poor OCT quality (motion/shadowing) that is associated with reduced bridge agreement.
- OCT-only acceptable: AMD staging and DME assessment are performed on OCT directly; DR staging is accepted only when the QC gate is satisfied (q≥0.8).
- Referral recommendation: any high-confidence pathology prediction (above the calibrated per-class threshold) combined with uncertainty signals (near-threshold probabilities or QC failure) triggers referral for confirmatory imaging and clinical adjudication.

### 5.3. Post-Deployment Auditing over Time

To ensure longitudinal safety, all predictions are logged together with calibrated probabilities, the QC score *q*, scanner metadata, and the final clinician-confirmed diagnosis (when available). We recommend conducting periodic audits (monthly or quarterly) to assess the following: (i) the deferral rate (q<0.8), (ii) stage-boundary confusion patterns, and (iii) indicators of drift, such as distribution shifts in embeddings or confidence levels. This monitoring process allows us to update thresholds, refine the QC gate, and initiate targeted re-training when systematic failure modes are detected.

## 6. Conclusions

The primary clinical promise of this work is to enable comprehensive, multi-label staging of retinal comorbidities. It specifically aims to bridge the gap between OCT-based structural analysis and fundus-based DR staging, using solely OCT imaging trained with unpaired data. The hierarchical modular architecture of the HMS system, enhanced by a cross-modality latent bridge, achieves high accuracy (macro-F1 = 0.989±0.006, micro-F1 = 94±0.003) and calibration (ECE = 2.1±0.4%) for multi-target classification and staging of AMD, DR, and DME. Notably, this system can perform DR staging using only OCT images via a contrast-aligned latent bridge, eliminating the need for fundus images in 96.1% of cases when specific quality control criteria are met. However, the remaining failure modes are mostly related to peripheral signs visible in fundus images that fall outside the macular OCT field of view.

Additionally, optimizing the F1 threshold for specific classes addresses class imbalance and outperforms traditional monolithic CNNs in macro-AUROC and calibration, while maintaining comparable accuracy. Cross-scan validation showed moderate robustness, with an accuracy of 86.1% for the Avanti XR to REVO NX direction, and 74.7% for the reverse direction. Furthermore, there was a systematic performance improvement of 7.2–7.3 p.p. over EfficientNet-B0. However, an absolute performance drop of 15.7 p.p. highlights the need for further adaptations to account for domain shifts.

## Figures and Tables

**Table 1:** Label harmonization across sources applied in the research.

Source	Native Labels (Used)	Mapping to Unified Label Space (This Paper)
In-house OCT	AMD stage (AREDS-based), plus clinical labels for DR/DME/NORM	Parent OCT labels: AMD = 1 if any AREDS stage; DR = 1 if DR present; DME = 1 if DME present; NORM = 1 if no pathology
OCTID (OCT-only)	Disease-level OCT categories (e.g., Normal, DR)	Parent OCT labels only: Normal → NORM; DR → DR; not used for DR staging
OCT–fundus dataset	Fundus DR stage (ICDR-based); OCT diagnostic classes	Fundus DR staging: Mild/Moderate/Severe NPDR, PDR; Parent OCT labels: NORM/AMD/DR/DME as provided

**Table 2:** Failure taxonomy for OCT-only DR staging (fundus grading is correct, OCT-only staging may be wrong) and its relation to missing retinal field-of-view (FOV) information.

Failure Category	Fundus-Visible Evidence (ICDR-Relevant)	Why OCT-Only May Miss It (FOV/Visibility)	Typical Error & Mitigation
Peripheral proliferative signs	Neovascularization away from the macula (e.g., NVE/NVD), peripheral hemorrhages	Standard macular OCT does not cover peripheral retina; the lesion may be outside the scanned area (missing FOV)	Under-staging (PDR → severe/moderate). Mitigation: acquire fundus or wider-field imaging; defer if QC fails.
Severity driven by lesion burden across fields	Stage boundaries depend on lesion extent across multiple retinal regions (counting-based rules)	OCT B-scans sample limited regions; disease burden outside sampled locations is unobserved (missing FOV)	Moderate↔severe confusion. Mitigation: request fundus/UWFI for confirmation in borderline cases.
Subtle early fundus signs	Single/few microaneurysms and small hemorrhages	These signs are more reliably assessed en-face on fundus; OCT-only may show weak or ambiguous correlates	Under-staging (mild → no DR). Mitigation: conservative routing/deferral for low-confidence cases.
Image-quality-driven errors	Fundus has sufficient quality, but OCT has artifacts (motion, shadowing, low signal)	Artifacts corrupt structural cues and degrade cross-modal projection quality	Unstable staging. Mitigation: quality control and manual verification when QC is triggered.
Borderline/subjective boundaries	Cases close to ICDR thresholds even for experts	Small differences in captured evidence across modalities amplify ambiguity	Stage flip near boundary. Mitigation: defer to fundus when clinically consequential.

## Data Availability

The proprietary clinical OCT dataset acquired at the Optimed Laser Vision Restoration Center (Ufa, Russia) is not publicly available due to privacy restrictions and institutional data governance requirements. The publicly available datasets used in this study are: (i) OCTID: Optical Coherence Tomography Image Database, available at https://doi.org/10.48550/arXiv.1812.07056 (accessed on 3 January 2026) [OCTID database link is provided by the authors in the associated arXiv record]; and (ii) OCT-AND-EYE-FUNDUS-DATASET, available at https://github.com/Traslational-Visual-Health-Laboratory/OCT-AND-EYE-FUNDUS-DATASET (accessed on 3 January 2026).

## Supplementary Materials

The following supporting information can be downloaded at: https://www.mdpi.com/article/10.3390/jimaging12010036/s1, Figure S1: Scatterplot of cosine similarity versus Fundus consistency for the cross-modal bridge for determining DR stages; Figure S2: Calibration curve and contributions to Expected Calibration Error (ECE); Table S1: Dataset Parameters; Table S2: Characteristics of the experimental dataset with class distribution and statistical parameters; Table S3: Comparison of backbone architectures for the parent model (multi-label classification on OCT); Table S4: Comparison of threshold calibration methods; Table S5: Multi-objective performance metrics of the parent model on the test sample; Table S6: Multi-objective metrics of the AMD model on the test sample; Table S7: Multi-objective metrics of the Fundus model on the test sample; Table S8: Confusion Matrices; Table S9: Progress of Contrastive Learning; Table S10: Cross-modal Bridge Learning Progress; Table S11: Ablative Study; Table S12: Claim-matched Competitive Baselines; Table S13: Cross-modal Discrepancy Analysis; Table S14: Sensitivity Analysis; Table S15: Calibration Comparison; Table S16: Computational efficiency; Table S17: Cross-scan validation; Table S18: Leave-One-Source-Out (LOSO) Generalization; Table S19: Audit of AMD and DR Co-activation Frequency in Test Data.
