# Supplementary material for: A Hierarchical Deep Learning Architecture for Diagnosing Retinal Diseases Using Cross-Modal OCT to Fundus Translation in the Lack of Paired Data
Source: J Imaging. 2026 Jan 8;12(1):36. doi: 10.3390/jimaging12010036 (PMC12842718; doi:10.3390/jimaging12010036)
Supplement: Supplementary file 1 [file jimaging-12-00036-s001.zip › jimaging-4042548-supplementary/supplementary.pdf]

Supplementary Materials: A Hierarchical Deep Learning Architecture for Diagnosing Retinal Diseases Using Cross-Modal OCT to Fundus Translation in the Lack of Paired Data

Ekaterina A. Lopukhova, Gulnaz M. Idrisova, Timur R. Mukhamadeev, Grigory S. Voronkov, Ruslan V. Kutluyarov and Elizaveta P. Topolskaya

Section S4.1: Parent Model Architecture and Loss Functions

Let $x \in \mathbb{R}^{1\times H\times W}$ be the input OCT image. The function f_{backbone} computes the embedding $z = f_{\text{backbone}}(x) \in \mathbb{R}^{d_{\text{emb}}}$, which is then L2-normalized to enhance the metric comparability in the latent space.

Next, the linear head $h_{\text{cls}} : \mathbb{R}^{d_{\text{emb}}} \to \mathbb{R}^4$ generates logits $s = h_{\text{cls}}(z)$. We apply the sigmoid function $\sigma(\cdot)$ to each logit to obtain independent probabilities for the four labels, resulting in $p = \sigma(s) \in \mathbb{R}^4$. This "backbone + head" composition is standard for multi-target classification with sigmoid outputs and ensures that probabilities are compatible and additive across tasks [1].

To enhance clinical consistency and align the model's output with hierarchical logic (NORM is mutually exclusive with pathologies), two penalties were added to the base Focal-BCE loss function. The resulting parent loss function is:

$$\mathcal{L}_{\text{Focal-BCE}} = -\frac{1}{B}\sum_{i=1}^{B}\sum_{j=1}^{4}\Big[y^s_{ij}\log(\sigma(o_{ij})) + (1-y^s_{ij})\log(1-\sigma(o_{ij}))\Big] \quad \text{(S1)}$$

The first penalty $\mathcal{R}_{\text{NORM}}$ minimizes the likelihood of joint activation of NORM with any pathology, which can be formally expressed as:

$$\mathcal{R}_{\text{NORM}} = \mathbb{E}\left[p_{\text{NORM}} \cdot \left(1 - \prod_{c\in\{\text{AMD,DR,DME}\}}(1-p_c)\right)\right] \quad \text{(S2)}$$

or, in a simpler linear approximation, $\mathcal{R}'_{\text{NORM}} = \mathbb{E}[p_{\text{NORM}} \cdot (p_{\text{AMD}} + p_{\text{DR}} + p_{\text{DME}})]$, which directly penalizes the simultaneous confidence in the absence of diseases and any pathology.

The second penalty $\mathcal{R}_{\text{co}} = \mathbb{E}[p_{\text{AMD}} \cdot p_{\text{DR}}]$ is designed to reduce the co-activation of AMD and DR. This measure is a dataset-specific heuristic introduced because there are few, if any, clear cases of AMD and DR co-labeling in the original sample. Its purpose is to prevent inconsistent decisions during the deployment phase of the current system version.

From a practical perspective, it is advisable to reduce the weight λ_{co} as we gather more reliable examples of comorbidity between AMD and DR. This adjustment will allow the model to learn joint patterns without introducing artificial decorrelation. Additionally, it is important to maintain $\mathcal{R}_{\text{NORM}}$ to ensure the clinical consistency of predictions distinguishing between "normal" and "pathological" cases.

To combine the advantages of binary cross entropy for multi-objective problems with a focusing factor that increases the contribution of "difficult" examples when classes are imbalanced, Focal BCE with label smoothing is used; let $p_{ij} = \sigma(o_{ij})$, where o_{ij} is the logit, σ is the sigmoid, and the smoothed labels are defined as

$$y^{\alpha}_{ij} = (1-\alpha)\cdot y_{ij} + \alpha/2 \quad \text{(S3)}$$

for $\alpha \in (0,1)$. Then, the class-weighted Focal BCE for batch B and four labels is written as:

$$L_{\text{Focal-BCE}} = -\frac{1}{B}\sum_{i=1}^{B}\sum_{j=1}^{4}\alpha_j\Big[(1-p_t)^{\gamma} y_{ij}^{\alpha}\log(\sigma(o_{ij})) + p_t^{\gamma}(1-y_{ij}^{\alpha})\log(1-\sigma(o_{ij}))\Big] \tag{S4}$$

where $\gamma \geq 0$ is the focusing parameter ($\gamma = 0$ yields the usual BCE), and $\alpha_j \in (0,1)$ are the class balancing coefficients that compensate for the imbalance of positives and negatives for the j-th label. In practice, it is more stable to calculate the BCE with logits, which is equivalent to using the sigmoid inside the error function and reduces numerical instability; here, the focal factor is introduced as a weight to the BCE terms, which coincides with the canonical formulation [2] in the binary case.

Since the optimal threshold for binary classification is rarely 0.5, especially in cases of class imbalance, we implemented a threshold calibration procedure. All decision thresholds were selected only on the validation split (i.e., on the validation fold within cross-validation, when applicable). The held-out test split/fold was never used to optimize thresholds or any calibration hyperparameters. All reported test metrics were computed by applying the thresholds obtained from the corresponding validation split/fold.

For each of the four classes in the validation set, we selected a threshold that maximized the F1-score. This approach resulted in a set of asymmetric thresholds, achieving an optimal balance between precision and recall for each diagnosis. To accomplish this, we employed class-specific threshold calibration to optimize the F1-score for each class in the parent model (per class) [3]:

$$\theta_j^* = \arg\max_{\theta\in[0.1,0.9]} F1_j(\theta) \tag{S5}$$

where $F1_j(\theta)$ represents the F1-score of class j at a given threshold θ. Theoretically, for calibrated probabilities, there is a relationship between the optimal F1-score and the threshold. In specific cases, the optimal threshold is half of the optimal F1-score. It highlights the importance of explicitly tuning θ when using F1 as the target metric. In practice, robust validation of per-class thresholds or the use of Bayesian or heuristic approximations to handle imbalanced data is often employed.

Section S4.2: Child Models and Cross-Modal Bridge

To refine the diagnosis of AMD into five stages, we employ a hybrid feature fusion scheme. This method combines local features from a CNN with global context obtained from a parent model and the prototypical geometry of the latent space. This approach aligns with multimodal/multi-source feature fusion practices, which have been shown to enhance quality by effectively integrating heterogeneous features [4].

We represent the local embedding from the specialist model as $\mathbf{z}_{\text{child}} \in \mathbb{R}^{d_{\text{emb}}}$, the global embedding from the parent model as $\mathbf{z}_{\text{parent}} \in \mathbb{R}^{d_{\text{emb}}}$, and the vector of distances to the class prototypes from the parent model $\mathbf{d}_{\text{centroids}} \in \mathbb{R}^{d_{\text{emb}}}$, where $d_c = \|\mathbf{z}_{\text{parent}} - \boldsymbol{\mu}_c\|_2$. The joint feature is constructed by concatenating $[\mathbf{z}_{\text{child}}, \mathbf{z}_{\text{parent}}, \mathbf{d}_{\text{centroids}}]$ and then fed into a MLP $g(\cdot)$.

The decision rule for classifying AMD into five stages is as follows:

$$\hat{y} = \arg\max \text{softmax}(g([\mathbf{z}_{\text{child}}, \mathbf{z}_{\text{parent}}, \mathbf{d}_{\text{centroids}}])) \tag{S6}$$

where softmax gives the distribution over the stages of AMD; $\boldsymbol{\mu}_c$ is the prototype (centroid) of class c. The addition $\mathbf{d}_{\text{centroids}}$ implements prototypal regularization by in-

troducing global geometric constraints on the feature space into the solution, which relies on prototype representations and "center loss" to improve inter-class separability and intra-class compactness [5].

The class prototype $\boldsymbol{\mu}_c$ is calculated as the average embedding of the parent across all examples where the label c is active. In the multi-label case, this is the "one vs. rest" average of positive instances for the class:

$$\begin{aligned} \boldsymbol{\mu}_c &= \frac{1}{|S_c|} \sum_{i \in S_c} \mathbf{z}^{(i)}_{\text{parent}}, \\ S_c &= \{i : y_c^{(i)} = 1\} \end{aligned} \tag{S7}$$

where S_c represents the set of indices for samples belonging to class c. The use of distances d_c as features or regularizers falls under the category of prototypical methods, such as Prototypical Networks and center regularizers like Center Loss. These techniques enhance the geometry of representations and improve resistance to class imbalance by explicitly aligning with each class's center. It has been validated both theoretically and empirically in recognition tasks [6].

The key innovation involves transferring knowledge about DR stages from fundus images to OCT using a two-step process. First, we perform a preliminary alignment of the contrast in the latent spaces, then train a regression "bridge." This method aligns with modern practices for cross-modality representation and significantly improves efficiency, particularly when annotated image pairs are limited. By using fundus images—the "gold standard" for DR staging—while only having access to OCT, we ensure that decisions remain semantically consistent across both modalities [7].

Let us denote the modality encoders as f_{oct} and f_{fun}, along with the projection heads as g_{oct} and g_{fun}. The normalized projections can be defined as:

$$\mathbf{p}_i^{\text{oct}} = \frac{g_{\text{oct}}(f_{\text{oct}}(x_i^{\text{oct}}))}{\|g_{\text{oct}}(f_{\text{oct}}(x_i^{\text{oct}}))\|_2}, \quad \mathbf{p}_i^{\text{fun}} = \frac{g_{\text{fun}}(f_{\text{fun}}(x_i^{\text{fun}}))}{\|g_{\text{fun}}(f_{\text{fun}}(x_i^{\text{fun}}))\|_2} \tag{S8}$$

where i indexes positive OCT–Fundus pairs. The cosine proximity is defined as:

$$\text{sim}(\mathbf{u}, \mathbf{v}) = \frac{\mathbf{u}^\top \mathbf{v}}{\|\mathbf{u}\|_2 \|\mathbf{v}\|_2} \tag{S9}$$

The temperature parameter $\tau > 0$ controls the "sharpness" of the softmax distributions, which influences the impact of complex negative samples. The foundational contrastive objective is implemented using NT-Xent, a widely accepted standard for cross-modal systems such as CLIP. NT-Xent maximizes the recognition accuracy of positive examples from a set of candidates while also providing a lower bound on the mutual information between the representations of the two modalities [8,9].

For bidirectional alignment, a symmetric version of NT-Xent using normalized projections and a cosine measure is employed, averaging the "image→fundus" and "fundus→image" paths:

$$\begin{aligned} \mathcal{L}_{\text{NT-Xent}} = -\frac{1}{2B} \sum_{i=1}^{B} \Bigg[&\log \frac{\exp(\text{sim}(\mathbf{p}_i^{\text{oct}}, \mathbf{p}_i^{\text{fun}})/\tau)}{\sum_{j=1}^{B} \exp(\text{sim}(\mathbf{p}_i^{\text{oct}}, \mathbf{p}_j^{\text{fun}})/\tau)} \\ &+ \log \frac{\exp(\text{sim}(\mathbf{p}_i^{\text{fun}}, \mathbf{p}_i^{\text{oct}})/\tau)}{\sum_{j=1}^{B} \exp(\text{sim}(\mathbf{p}_i^{\text{fun}}, \mathbf{p}_j^{\text{oct}})/\tau)} \Bigg] \end{aligned} \tag{S10}$$

where $\mathbf{p}^{\text{oct}}$ and $\mathbf{p}^{\text{fun}}$ represent the outputs of the projection networks, the term sim(,) denotes the cosine similarity between these outputs, B denotes the batch size, and negative examples are selected from the other elements in the batch, following a standard approach

used in methods such as SimCLR and CLIP. In essence, InfoNCE serves as a contrastive objective that mathematically maximizes the lower bound on the mutual information between the representations of two modalities. This framework compels encoders to maintain invariant common information, which has been shown to improve transfer performance in various deep learning tasks.

In practice, the quality of contrastive learning depends heavily on the number and diversity of negatives; SimCLR empirically benefits from larger batches, but this is limited by GPU memory. The momentum encoder parameters are updated using a rule $\theta_{\text{key}} \leftarrow m\theta_{\text{key}} + (1-m)\theta_{\text{query}}$ with $m \approx 0.999$, ensuring temporal consistency of features in the queue and improving training stability. Furthermore, research shows that "difficult" negatives are particularly useful, and increasing their proportion through larger dictionaries/queues improves the quality of representations and proximity retrieval [10].

After embedding alignment, the "exact transformation" problem is addressed by a small MLP (Multi-Layer Perceptron) bridge, denoted as h. This bridge predicts $\hat{\mathbf{z}}^{\text{fun}} = \frac{h(\mathbf{z}^{\text{oct}})}{\|h(\mathbf{z}^{\text{oct}})\|_2}$ in the encoder's unnormalized space from $\mathbf{z}^{\text{oct}} = f_{\text{oct}}(x^{\text{oct}})$ simplifying the task significantly after pre-alignment via a contrastive stage [11]. The primary goal of the bridge is to perform a combined regression in the feature space while achieving geometric alignment. This process aims to simultaneously minimize the Euclidean error and maximize the cosine similarity in the target latent space [7]. Intuitively, pre-contrast alignment reduces the nonlinearity of the mapping problem, improving the sampling efficiency of the bridge. This improvement is confirmed by the performance of bidirectional cross-modal systems trained with symmetric contrastive learning [12].

The architecture includes a multi-component loss function:

$$L_{\text{bridge}} = \lambda_{\text{MSE}} L_{\text{MSE}} + \lambda_{\text{cos}} L_{\text{cosine}} + \lambda_{\text{KL}} L_{\text{KL}} + \lambda_{\text{NT-Xent}} L_{\text{NT-Xent}} + \lambda_{\text{MMD}} L_{\text{MMD}} \tag{S11}$$

In this context, $\lambda_{\text{cos}} L_{\text{cosine}}$ represents the weighting coefficient for the cosine component of the loss function used for normalized representations. When paired OCT-Fundus data is unavailable, the system switches to a backup training mode. In this mode, missing paired data is replaced with fundus embeddings, approximated as a weighted mixture of pre-computed class prototypes in the fundus representation space:

$$z_{\text{bridge}}^{\text{fallback}} = \sum_{k=1}^{K} \alpha_k p_k^{\text{fundus}} \tag{S12}$$

where α_k are the attention weights calculated based on the proximity of the embedding OCT to the centroids of the parent model.

The Fundus model classifies DR stages according to the international classification of NPDR into four classes: MILD_NPDR, MODERATE_NPDR, SEVERE_NPDR, and PDR. The architecture is based on a convolutional encoder similar to the parent model, with an adaptation for grayscale images. A key element is the system of class prototypes, calculated as the centroids of the embeddings for each class:

$$p_k^{\text{fundus}} = \frac{1}{|T_k|} \sum_{i \in T_k} z_{\text{fundus}}^{(i)} \tag{S13}$$

where T_k is the set of samples of the class k in the fundus image data, p_k^{fundus} is the prototype of the k-th class.

The entire system is trained in stages to ensure the stability and high quality of each component:

1. Parent and Fundus Model Training: Initially, the base models are trained independently for feature extraction.
2. Contrastive Alignment: Next, projection networks are trained using fixed backbone weights.
3. Bridge Training: The final stage combines information from all the previously trained models.

The Results chapter outlines the step-by-step training process for the entire system and presents the outcomes. We utilized a dataset that included both our own clinical data and publicly available datasets. This approach allowed for comprehensive coverage of various pathological conditions across both modalities and facilitated the collection of paired OCT and fundus images.

Section S4.3: Dataset Characteristics

The experimental dataset is a comprehensive multimodal collection of medical images consisting of 8,159 images across two primary ophthalmic imaging modalities. The dataset's structure reflects clinical reality, with 4,047 OCT images, each annotated with detailed multi-label information, and 4,112 fundus images. A notable aspect of this dataset is the limited but significant collection of paired OCT-fundus images: only 128 pairs, accounting for 3.1% of the total dataset. This scarcity of strictly paired multimodal data is common in medical research. Since our approach relies on an strictly unpaired training paradigm (zero paired samples used for optimization), the 128 available paired samples serve exclusively as a test set.

The dataset was compiled from three sources to ensure comprehensive coverage of various pathological conditions, contributing to a diverse range of diagnoses. By integrating these data sources, we can gain a thorough understanding of all stages of the diseases studied, including AMD, DR, and DME, using OCT methods. For DR, staging was presented using fundus imaging.

The first source is a proprietary clinical dataset obtained from the Optimed Laser Vision Restoration Center in Ufa, Russia. This dataset contains 2,185 OCT images acquired with two spectral-domain tomographic systems: the Avanti XR (Optovue; USA) and the REVO NX (Optopol; Poland). This selection ensures that the data is representative of different imaging technologies. Each OCT image was classified according to the Age-Related Eye Disease Study (AREDS) standards, which are used in international clinical trials to stage AMD. The dataset includes comprehensive information on AMD, covering all five stages of the disease as defined by the AREDS classification [13].

The second data source is the open Optical Coherence Tomography Image Database (OCTID), developed by the University of Waterloo [14]. This database includes over 500 high-quality OCT images categorized by various pathological conditions, such as AMD, central serous retinopathy, DR, and macular holes. For the experimental dataset, we selected retinal cases free of pathologies and diabetic retinopathy. The images were captured using raster scanning with a scan length of 2 mm and a resolution of 512×1024 pixels, ensuring high detail of retinal structures.

The third source is the OCT-AND-EYE-FUNDUS-DATASET, developed for the study of DME and DR. This dataset includes 1,548 fundus images and 1,113 macular OCT images collected between 2015 and 2022, supported by CONACYT grants CF-2019-1759 and PAPIIT IN 205420, along with assistance from the Mexican Institute of Ophthalmology, the Association for the Prevention of Blindness, and the Bajio Retina Institute [15]. All fundus images are standardized to 1000×1000 pixels, while the OCT images are 1408×573 pixels, and all images are presented in JPG format. A unique feature of this dataset is the paired OCT-Fundus image nomenclature, which allows for identification of the patient, the

eye being examined (right or left), the image modality, and the sample number. Certified retinologists and ophthalmologists diagnosed all cases.

The dataset's diagnostic structure includes four main disease classes: no abnormalities (normal), AMD, DR, and DME. To ensure detailed diagnostics, five AMD subclasses and four DR subclasses are provided, as presented in Table S1. AMD is classified into several stages based on specific characteristics.

1. Early Stage: This stage is defined by the presence of medium-sized drusen, which are less than 125 microns in diameter.
2. Intermediate Stage: In this stage, individuals have large drusen (greater than 125 microns in diameter) and/or pigmentary changes.
3. Late Atrophic Form (Late_AMD→Atrophy): This form is characterized by geographic atrophy, where there is a significant loss of retinal pigment epithelium.
4. Late Neovascular Form (Late_AMD→nAMD): Also known as "wet" AMD, this form involves pathological choroidal neovascularization, where newly formed blood vessels penetrate through Bruch's membrane. This condition is observed on OCT as subretinal and/or intraretinal fluid, pigment epithelial detachment, and fibrovascular proliferation.
5. Late AMD/Subretinal Fibrosis: This is the terminal stage of the exudative form of AMD, which occurs as a result of prolonged choroidal neovascularization. It leads to the formation of a disciform scar at the site of a previously active neovascular membrane.

The international classification criteria for DR are outlined in the ETDRS system, which initially included 14 severity levels. However, the ICDR classification simplifies this to five main stages: no diabetic retinopathy, mild nonproliferative diabetic retinopathy (MILD_NPDR), moderate nonproliferative diabetic retinopathy (MODERATE_NPDR), severe nonproliferative diabetic retinopathy (SEVERE_NPDR), and proliferative diabetic retinopathy (PDR). This classification was chosen for this study because it strikes a balance between ease of use and scientific validity [16].

The dataset exhibits a clinically realistic distribution of pathologies, with a natural class imbalance that reflects the epidemiology of retinal diseases. This imbalance is typical of medical datasets, where the prevalence of different disease stages varies significantly in clinical practice, requiring specialized learning methods to handle imbalanced data effectively [17].

Table S1. Dataset Parameters

Parameter	Value
Total images	8159
Modalities	2 (OCT, Fundus)
Primary diagnostic classes	4 (No abnormalities, AMD, DR, DME)
Detailed AMD subclasses	5
Detailed DR subclasses	4
Imbalance ratio (max/min)	91.8 (2203/24)
Paired OCT-Fundus data	3.1%
Image size (preprocessed)	512 × 512 pixels

To ensure a correct assessment of the model's generalization ability, all experiments were conducted using 5-fold cross-validation with a fixed split by patient identifiers. This strategy is critical in medical imaging applications, as images of the same patient can exhibit high correlation, and random distribution between the training and test sets would lead

to overestimation of the model's performance and a loss of generalization to new patients [18]. The composition of the whole dataset is presented in Table S2.

Table S2. Characteristics of the experimental dataset with class distribution and statistical parameters

Modality	Disease/Stage	Subclass	N	% Class	% Modal.
OCT	AMD	Early	219	10.0	5.4
		Intermediate	905	41.4	22.4
		Late/Atrophy	172	7.8	4.3
		Late/Neovascular	666	30.5	16.5
		Late/Fibrosis	223	10.2	5.5
	**AMD Subtotal**		**2185**	**100.0**	**54.0**
	DR	Unmarked	186	71.0	4.6
		NPDR	52	19.8	1.3
		PDR	24	9.1	0.6
	**DR Subtotal**		**262**	**100.0**	**6.5**
	**DME**		**314**	**100.0**	**7.8**
	**No abnormalities**		**1286**	**100.0**	**31.8**
	**OCT Total**		**4047**		**100.0**
Fundus	DR	Mild NPDR	370	9.0	9.0
		Moderate NPDR	999	24.3	24.3
		Severe NPDR	193	4.7	4.7
		PDR	347	8.4	8.4
		No pathologies	2203	53.6	53.6
	**Fundus Total**		**4112**	**100.0**	**100.0**
**GRAND TOTAL**			**8159**		

Section S4.4: Backbone Architecture Comparison

The dataset was split into training, validation, and test sets at 80/10/10. This approach aligns with standard practices in medical imaging, ensuring sufficient training data while preserving the representativeness of the validation and test sets. The loss function selected for this study was BCEWithLogitsLoss (binary cross-entropy with logits) [19,20].

The results of a comparative analysis of four encoder architectures are displayed in Table S3. The metrics shown are averaged over five random initializations to ensure the statistical reliability of the findings. The test sample consisted of 578 images, categorized as follows: NORM (no pathology) = 136; AMD = 289; DR = 101; and DME = 50. The latency (input delay) was measured on an NVIDIA RTX 3060 GPU with a batch size of 16 and using FP32 floating-point precision.

Table S3. Comparison of backbone architectures for the parent model (multi-label classification on OCT)

Architecture	Macro-F1	Micro-F1	Hamming	Jaccard	Params (M)	Lat. (ms)
ResNet18	0.989 ± 0.006	0.994 ± 0.003	0.002 ± 0.000	0.996 ± 0.001	11.7	13.7
ResNet34	0.987 ± 0.005	0.993 ± 0.002	0.002 ± 0.000	0.996 ± 0.001	21.8	18.2
EfficientNet-B0	0.991 ± 0.007	0.995 ± 0.003	0.002 ± 0.001	0.996 ± 0.002	5.3	16.5
ConvNeXt-Tiny	0.938 ± 0.031	0.952 ± 0.021	0.009 ± 0.002	0.953 ± 0.008	28.6	21.3

The EfficientNet-B0 architecture achieved the best discriminatory performance among the models tested, with a Macro-F1 score of 0.991 and a Jaccard index of 0.996. It outperformed ResNet18 by 0.2 percentage points in Macro-F1, though the difference is statistically insignificant ($p > 0.05$ given the 95% confidence interval overlap). In contrast, the ConvNeXt-Tiny architecture showed significantly poorer results, with a Macro-F1 score of 0.938. It indicates convergence issues when training on limited medical data, a com-

mon challenge for modern architectures with many parameters and a scarcity of training examples.

A key advantage of EfficientNet-B0 is its optimal balance between classification performance and computational efficiency. The model has 5.3 million parameters and a latency of just 16.5 ms. It is significantly lower than ResNet18 (11.7 million parameters), ResNet34 (21.8 million parameters), and especially ConvNeXt-Tiny (28.6 million parameters). These features make EfficientNet-B0 the ideal choice for clinical deployment in environments with limited computational resources, where high diagnostic accuracy is essential and minimal image processing latency is needed.

Section S4.5: Threshold Calibration Methods

In a multi-objective setting, optimizing class-specific thresholds is essential to address class imbalance [21]. The comparison of four calibration strategies, as shown in Table S4, highlights the effectiveness of the F1-measure optimization approach:

Table S4. Comparison of threshold calibration methods. (Thresholds are selected on the validation split/fold only; test split/fold is never used for threshold selection)

Method	NORM	AMD	DR	DME	Macro-F1	Micro-F1	Hamming
Default 0.5	0.50	0.50	0.50	0.50	0.923	0.945	0.0089
Youden Index	0.32	0.18	0.71	0.82	0.976	0.983	0.0031
F1-optimized	0.29	0.15	0.67	0.78	0.989	0.994	0.0018
Prec-Rec bal.	0.35	0.22	0.69	0.80	0.981	0.987	0.0024

F1-optimized thresholds provide the best balance between Precision and Recall for each class while also accounting for clinical priorities. The Youden index combines these characteristics into a single metric, helping to determine the optimal cutoff point. However, in multi-objective classification with imbalanced classes, optimizing the F1-score for each class yields better results. The approach includes a more aggressive detection strategy for AMD, with a threshold of 0.15 to minimize the risk of missing late-stage disease, during which vision loss is particularly likely. In contrast, a more conservative strategy is adopted for DME, with a threshold of 0.78 to avoid unnecessary interventions in patients with significant comorbidities [22]. The optimal thresholds identified were: NORM=0.29, AMD=0.15, DR=0.67, DME=0.78. These thresholds reflect the need to address the class imbalance in the dataset, where AMD constituted more than 50% of the OCT samples. In comparison, DME accounted for only 8.6% of the examples.

Section S4.6: Parent Model Performance Metrics

A comprehensive analysis of the model's performance for each class is presented in Table S5. This table displays the Precision, Recall, and F1-scores, along with their respective confidence intervals. The confidence intervals were calculated using the bootstrap method with 1,000 iterations, providing a statistically sound estimate of the uncertainty for each metric. This approach aligns with contemporary practices for validating machine learning models [23].

Table S5. Multi-objective performance metrics of the parent model on the test sample

Label	Precision	Recall	F1-Score	N examples
NORM	0.993 ± 0.004	1.000 ± 0.000	0.996 ± 0.002	136
**AMD**	$\mathbf{1.000 \pm 0.000}$	$\mathbf{1.000 \pm 0.000}$	$\mathbf{1.000 \pm 0.000}$	289
DR	0.995 ± 0.006	0.990 ± 0.008	0.992 ± 0.005	101
DME	0.990 ± 0.010	0.978 ± 0.015	0.984 ± 0.011	50

The AMD classification model demonstrates ideal performance, achieving precision, recall, and F1-score values of 1.00. This outstanding performance is attributed to its dominance within the dataset and the clear morphological features observed on OCT, such as drusen and retinal pigment epithelial detachment. The model employs a low classification threshold of 0.15, enabling aggressive detection of even the earliest manifestations of AMD, thereby minimizing false negatives and facilitating timely identification of disease progression. Based on the EfficientNet-B0 architecture, this model efficiently extracts features from medical images through transfer learning and an optimized convolutional layer structure [3,24].

The normal range (NORM) achieves a recall rate of 1.00, which means that no cases are missed. It is essential for minimizing false alarms among healthy patients during screenings. Additionally, the precision is 0.993, indicating one false positive per 136 cases. This approach aligns with clinical practices that prioritize a conservative screening strategy to reduce the risk of overdiagnosis.

The DME class has a completeness of 0.978, indicating that one case is missed for every 50. It is primarily due to borderline cases of minimal intraretinal fluid, which lie at the edge of the clinical criteria for DME with central involvement [25].

The DR class has a recall of 0.990, with two false-negative results corresponding to MILD_NPDR, which includes a single microaneurysm. This scenario is attributed to OCT's limited informativeness for DR staging, as microaneurysms are less accurately visualized in OCT images [3,26].

Section S4.7: AMD Staging Module Performance

The specialized module developed for diagnosing AMD showed impressive diagnostic performance, achieving an overall accuracy of 98.3 ± 1.4%. This high level of performance can be attributed to the effective integration of local features extracted by the CNN with the global context provided by the parent model. Additionally, the use of prototypical geometry in the latent space enhances the discrimination between the four stages of AMD. This approach allows the system to differentiate between classes by forming class prototypes as vectors in the latent space and calculating the Euclidean distances between the image representations and these prototypes.

Table S6. Multi-objective metrics of the AMD model on the test sample

AMD Class	Precision	Recall	F1-Score	N samples
Early	0.967 ± 0.018	0.906 ± 0.025	0.936 ± 0.019	32
Intermediate	0.979 ± 0.012	**0.992 ± 0.008**	**0.985 ± 0.010**	126
**Late/Atrophy**	**1.000 ± 0.000**	**1.000 ± 0.000**	**1.000 ± 0.000**	26
**Late/Fibrosis**	**1.000 ± 0.000**	**0.994 ± 0.012**	**0.991 ± 0.008**	23
**Late/nAMD**	**1.000 ± 0.000**	0.991 ± 0.009	0.995 ± 0.005	110

As shown in Table S6, Intermediate AMD achieves high performance metrics, with an F1-score of 0.985, indicating a strong balance between accuracy and recall. In the later stages of the disease, recognition accuracy is nearly flawless, with both atrophy and nAMD achieving F1-scores of 1.00 and 0.995, respectively. This high accuracy is crucial for the timely administration of anti-VEGF therapy for neovascular disease [27].

Three cases of early AMD were misclassified as intermediate AMD, accounting for 9.4% of all early AMD cases. These misclassifications occurred because the drusen size was borderline, close to the 125-micron threshold used by the AREDS system to differentiate between early and intermediate stages of the disease.

According to the presented metrics, the LATE_AMD_FIBROSIS class has demonstrated a reliable ability to identify end-stage fibrosis, with only a few false negatives. Specifically,

there was one false-negative result out of 23 cases (1/23, 4.3%) involving a borderline case with partial organization of fibrovascular tissue that visually overlapped with signs of active neovascularization. This outcome can be attributed to the gradual transition from the exudative nAMD to the fibrotic stage throughout the long-term progression of the disease. The class demonstrated a high recognition accuracy, with a Precision of 1.000, indicating no false-positive predictions. It minimizes the risk of prematurely discontinuing active antiangiogenic therapy.

Section S4.8: DR Staging Module Performance

As shown in Table S7, the developed model for classifying DR stages using fundus images demonstrated an overall accuracy of 94.8 ± 0.9%.

Table S7. Multi-objective metrics of the Fundus model on the test sample

Class	Precision	Recall	F1-Score	N samples
Mild NPDR	0.907 ± 0.019	0.925 ± 0.021	0.916 ± 0.018	53
**Moderate NPDR**	**0.957 ± 0.015**	**0.975 ± 0.012**	**0.966 ± 0.011**	160
Severe NPDR	0.929 ± 0.023	0.929 ± 0.025	0.929 ± 0.022	28
PDR	0.976 ± 0.018	0.889 ± 0.028	0.930 ± 0.024	45

The model identified five cases of misclassifying proliferative PDR as moderate/severe NPDR, representing (5/45, 11.1%) of the 45 cases analyzed. This misclassification occurred due to overlapping neovascular features and the inherent difficulty in detecting extra-disc neovascularization when the lesion area is less than half the optic nerve head area. Research indicates that identifying small neovascular epithelium (NVE) lesions poses significant diagnostic challenges, even with modern imaging techniques [28,29].

Moderate NPDR exhibits the highest classification performance, with an F1-score of 0.966. This strong performance is attributed to the clear diagnostic criteria defined by the ICDR for this stage. Moderate NPDR is characterized as being "more than just microaneurysms but less than severe NPDR." Specifically, it is identified by the presence of microaneurysms, intraretinal hemorrhages, or venous tortuosity, none of which reach the severity depicted in standard ETDRS images 2A (for hemorrhage/microaneurysm severity), 6A (for venous tortuosity), and 8A (for intraretinal microvascular abnormalities) [30,31]. This clear definition provides explicit diagnostic criteria that aid machine learning algorithms.

0.1. Error structure via confusion matrices (Parent, AMD staging, and DR staging)

Table S8 reports confusion matrices for (i) the parent diagnostic model, (ii) the 5-class AMD staging module, and (iii) the 4-class fundus DR staging module. Unlike aggregate scores, these matrices explicitly localize which classes/stages dominate the residual error and therefore quantify clinically expected ambiguity patterns.

The parent model shows near-ceiling performance on the held-out test set, with only isolated errors. The observed DR miss corresponds to the mildest DR presentation (single microaneurysm), which is known to be difficult to stage reliably from structural OCT alone. Similarly, the single DME miss represents a borderline fluid case, consistent with the clinical threshold nature of DME criteria. Overall, the confusion structure confirms that remaining parent-level errors are concentrated in subtle/borderline findings rather than in gross pathology recognition.

For AMD staging, the dominant confusion occurs between Early and Intermediate AMD. Specifically, 3 out of 32 Early AMD cases are predicted as Intermediate, which quantifies the ambiguity already discussed in the text and corresponds to borderline drusen

size around the AREDS threshold. Importantly, no non-adjacent stage confusions are observed (e.g., Early is not confused with late atrophy or neovascular AMD), indicating that the model preserves the ordinal clinical structure of AMD progression. Late stages (atrophy and neovascular AMD) remain highly separable due to distinctive OCT morphology, and the residual errors, when present, are limited to morphologically similar late subtypes.

For DR staging, most errors occur at stage boundaries, primarily Mild→Moderate NPDR and PDR→(Moderate/Severe) NPDR. In particular, 5 out of 45 PDR cases are predicted as Moderate/Severe NPDR, matching the known difficulty of detecting small neovascular lesions and borderline proliferative signs on color fundus images. This matrix-level view complements per-class precision/recall by highlighting that the residual error is not uniformly distributed, but concentrated around clinically subjective decision thresholds and visually overlapping criteria.

Table S8. Reconstructed Confusion Matrices for Parent, AMD, and DR Models on the Test Set. Values on the main diagonal represent correctly classified instances (True Positives).

(a) Parent Multi-Label Model (Diagnostic Classes)

True \Pred	NORM	AMD	DR	DME
**NORM (136)**	**136**	0	0	0
**AMD (289)**	0	**289**	0	0
**DR (101)**	1	0	**100**	0
**DME (50)**	0	0	1	**49**

Note: DR miss is Mild NPDR; DME miss is borderline fluid.

(b) AMD Staging Model (5 Classes)

True \Pred	Early	Inter	Late/Atr	Late/nAMD
**Early (32)**	**29**	3	0	0
**Inter (126)**	0	**126**	0	0
**Late/Atr (26)**	0	0	**26**	0
**Late/nAMD (110)**	0	0	0	**109**
**Late/Fibr (23)**	0	0	0	**1** / 22 (Correct)

Errors occur only between adjacent/morphologically similar stages.

(c) DR Staging Model (Fundus 4 Classes)

True \Pred	Mild	Mod.	Sev.	PDR
**Mild (53)**	**49**	4	0	0
**Mod. (160)**	3	**156**	1	0
**Sev. (28)**	0	2	**26**	0
**PDR (45)**	0	3	2	**40**

Section S4.9: Cross-Modal Bridge Training and Analysis

The contrastive alignment step for OCT and fundus images is carried out using the NT-Xent (Normalized Temperature-scaled Cross-Entropy) loss function. This process involves a pulse encoder with $m = 0.999$ and a negative queue containing 512 samples [11]. As outlined in Table S9, the training process demonstrates a two-phase dynamic characteristic of contrastive learning. During the first 20 epochs, the loss function on the training set decreases rapidly. A gradual improvement follows this in the Recall@1 metric, which measures the accuracy of retrieving the first-nearest neighbor. The maximum performance for Recall@1 is achieved at the 54th epoch.

Contrast learning applied to medical images demonstrates a specific convergence pattern. Over 54 epochs, the training loss declines sharply, from 8.07 to 0.66. This is paired with a gradual improvement in the quality of the learned representations, as evidenced by the Recall@1 metric, which rises from 0.016 to 0.411. The stabilization of the temperature parameter at approximately $\tau \approx 0.0507$ aligns with theoretical recommendations for contrastive learning, particularly when using small batch sizes. This temperature parameter

Table S9. Progress of Contrastive Learning

Epoch	Train Loss	Val Loss	Recall@1	Temp.
1	8.070	0.408	0.016	0.0500
7	7.203	0.329	0.266	0.0503
24	1.712	0.322	0.297	0.0508
54	0.662	0.310	**0.411**	0.0507
60	0.660	0.309	0.313	0.0507

is crucial as it regulates the distribution of gradients among positive and negative pairs. The peak Recall@1 value of 0.411 indicates a successful alignment of the latent-space geometries across the two modalities, facilitating robust cross-modal image retrieval [32].

There is still potential for further performance improvement by increasing the size of the negative queue or by implementing strategies for dynamically selecting complex negative samples, which have proven effective in medical imaging tasks [33].

The cross-modality bridge between OCT and fundus images was trained using a multi-component loss function combining seven regularization components: mean square error (MSE), cosine closeness (Cosine), Kullback-Leibler divergence (KL-divergence), InfoNCE contrastive loss, prototype loss (Prototype), maximum mean divergence (MMD), and correlation alignment (CORAL) [34–36]. The training process, as presented in Table S10, demonstrated stable convergence of all components and high knowledge transfer rates between modalities, consistent with state-of-the-art approaches to cross-modality representation learning in medical imaging.

Table S10. Cross-modal Bridge Learning Progress

Ep.	Cos Sim	Fund Cons	MSE	Cos Loss	KL	InfoNCE	Proto	MMD
1	0.7834	0.8147	0.0028	0.3562	0.3403	2.913	1.589	0.786
5	0.8286	0.8147	0.0013	0.1636	0.2208	2.759	1.475	0.308
10	0.8393	0.8147	0.0012	0.1497	0.2145	2.715	1.469	0.325
18	0.8427	0.8147	0.0011	0.1426	0.2154	2.654	1.470	0.327
30	**0.8451**	**0.9844**	0.0011	0.1367	0.1908	2.605	1.466	0.294

The MSE component ensures the accuracy of the Euclidean projection, achieving a final MSE of 0.0011. It indicates an efficient feature mapping between the representation spaces of the two modalities. The cosine loss maximizes angular similarity between representations, achieving a value of 0.1367. It is a standard approach in contrastive learning for aligning cross-modal embeddings. Furthermore, the contrastive InfoNCE loss helps maintain separation between positive and negative pairs, with a final value of 2.605. It creates a discriminative representation space and prevents representation collapse [37].

The Kullback-Leibler divergence is used to align probability distributions across different modalities, reducing it from 0.3403 to 0.1908. This decrease indicates a successful alignment of the statistical properties of the representations. Additionally, prototype loss encourages the clustering of semantically similar representations around shared prototypes and stabilizes at 1.466. This process enhances the model's ability to discriminate between classes. Furthermore, maximum mean discrepancy (MMD) and correlation alignment are utilized to adapt feature distributions across domains, with MMD decreasing from 0.786 to 0.294. This reduction signifies a successful convergence of representation distributions across different modalities.

The significant increase in the fundus consistency score from 0.815 to 0.984 between epochs 18 and 30 indicates a notable improvement in the bridge's ability to produce semantically consistent representations. This result shows that the multi-component learning strategy not only ensures the geometric alignment of feature spaces but also achieves a

high level of semantic correspondence between OCT and fundus imaging modalities. It is essential for accurate cross-modality diagnostics of retinal diseases [38].

An ablation study highlights the crucial role of contrastive alignment: removing the InfoNCE and cosine components results in the most significant decline in cosine similarity and fundus consistency. This finding aligns with results observed in contrastive learning for medical imaging and cross-modal feature alignment. The contribution of the prototype component is moderate but still important for effectively clustering rare classes. Meanwhile, the MSE, KL, MMD, and CORAL techniques ensure both geometric and statistical consistency of distributions, facilitating the transfer of knowledge between OCT and fundus modalities.

To evaluate the contribution of each component in the loss function outlined in Table S11, we systematically removed one component at a time while keeping the other settings constant. We then measured validation cosine similarity and consistency with fundus representations, which is a standard practice for analyzing multi-component targets in multimodal systems [39,40]. The underlined rows indicate the most significant degradations when the InfoNCE and cosine components were removed, highlighting the essential role of contrastive pressure in aligning representations within the common feature space.

Table S11. Ablative Study of Loss Function Components

Excluded Component	Cos Sim	Fund Agr	Δ Cos	Δ Agr
Full (baseline)	0.8451	0.9844	0.0000	0.0000
w/o MSE	0.8389	0.9687	-0.0062	-0.0157
**w/o Cosine**	**0.8124**	**0.9531**	**-0.0327**	**-0.0313**
w/o KL	0.8402	0.9766	-0.0049	-0.0078
**w/o InfoNCE**	**0.8156**	**0.9453**	**-0.0295**	**-0.0391**
w/o Prototype	0.8203	0.9609	-0.0248	-0.0235
w/o MMD	0.8398	0.9688	-0.0053	-0.0156

Removing InfoNCE (which had a loss of -0.0295 for cosine similarity) and the cosine component (with a loss of -0.0327) resulted in the highest overall loss. This finding highlights the critical importance of contrast and angular criteria for robust cross-modal alignment in medical tasks, particularly for improving predictions in ophthalmology. Additionally, the prototype loss contributed moderately but consistently, with a value of -0.0248. This contribution helps stabilize class geometry and enhances the structure of embeddings, aligning with previous findings on prototype-contrast learning for sparse and long-tailed classes.

The MSE and KL components maintain metric accuracy and distribution consistency, so excluding them results in a small but noticeable degradation in quality. Degradations from excluding MMD and CORAL are also expected, as these measures reduce domain-specific distribution divergence and improve feature transferability, as confirmed in reviews and applied adaptation schemes across visual domains [41,42].

Table S12 summarizes the two baselines that are explicitly aligned with the two central methodological claims of this paper: (i) calibrated multi-label OCT outputs and (ii) unpaired cross-modal transfer from OCT to the fundus latent space.

The purpose of this table is to make the attribution of gains transparent: improvements should not be conflated with non-comparable training/evaluation recipes, but instead linked to either calibration-aware multi-label learning or the proposed cross-modal alignment/bridge design.

A strong monolithic baseline for the multi-label claim is defined as a single OCT backbone with a sigmoid multi-label head, evaluated under the same calibration-aware

Table S12. Claim-matched competitive baselines reported in this work. The goal is to compare against baselines that match the two core claims: (i) calibrated multi-label outputs and (ii) unpaired cross-modal transfer.

Baseline (claim)	Definition	Where reported
Calibrated multi-label OCT baseline (claim: calibration + multi-label)	Monolithic sigmoid multi-label OCT classifier with calibration-aware evaluation (validation-only per-class threshold tuning; calibration assessed via ECE on held-out test).	SM Sec. 4.5 (Table SM S4) and SM Sec. 4.10 (Table S15).
Unpaired cross-modal baseline (claim: unpaired transfer)	Bridge variant without contrastive pressure (w/o InfoNCE), showing reduced cosine similarity / fundus agreement compared to the full objective.	SM Sec. 4.9 (Table S11).

protocol used in this work (validation-only per-class threshold tuning and ECE evaluation on the held-out test fold).

Accordingly, the key reference points for this baseline are the threshold calibration study (Table S4) and the calibration comparison on the held-out test set (Table S15, SM Sec. 4.10).

For the unpaired transfer claim, we use a degraded bridge variant without contrastive pressure (w/o InfoNCE), which serves as a practical unpaired baseline under the same data and evaluation protocol. As reported in the bridge ablation study (Table S11), removing InfoNCE consistently reduces cross-modal alignment quality (cosine similarity / fundus consistency), demonstrating that the proposed contrastive alignment component is a major driver of successful transfer rather than a minor auxiliary regularizer.

To assess the quality of cross-modality transfer between OCT and fundus representations, we need to identify instances where the bridge model fails to project OCT accurately into the fundus representation space. Conducting a discrepancy analysis allows us to establish quantitative agreement thresholds between OCT-based predictions and synthesized fundus representations. This analysis also helps us characterize common causes of projection failures, which enhances the transparency and interpretability of the hybrid diagnostic system. Identifying these cases is essential to developing a clinical quality control system that flags uncertain results and refers them for additional manual verification by a specialist.

To assess the quality of cross-modal transfer, we analyzed cases with low agreement between the OCT model and the cross-modal bridge's predictions. Table S13 presents representative examples of cases where the agreement was less than 0.8. These cases accounted for only 3.9% (5/128) of all paired data in the test set.

Table S13. Cross-modal Discrepancy Analysis

Case	OCT	Bridge	Cos Sim	Agr	Reason
C_0089	Severe NPDR	Mod. NPDR	0.712	0.625	Motion artifacts in OCT
C_0134	PDR	Severe NPDR	0.689	0.750	Uneven illumination
C_0267	Mild NPDR	Normal	0.634	0.375	Physiological variations
C_0356	Mod. NPDR	Mild NPDR	0.756	0.625	Borderline microaneurysm
C_0498	PDR	Mod. NPDR	0.723	0.750	Neovascular outside OCT field

The average Cosine Similarity for mismatches was 0.703 ± 0.041, significantly lower than the average successful transfer rate of 0.863 ± 0.028. It provides a reliable quantitative indicator of projection quality for clinical monitoring.

The established cosine similarity threshold of 0.8 ensures a high specificity for identifying problematic cases. Specifically, 96.1% (123/128) of cases showed agreement above this threshold, demonstrating the reliability of the cross-modality bridge in most clinical scenarios. For cases with a cosine similarity below 0.8, it is recommended to conduct additional manual analysis by a specialist or to acquire higher-quality images to enhance the reliability of the diagnostic conclusion.

We use cosine similarity between the projected OCT embedding and the reference fundus embedding as a lightweight quality-control (QC) indicator for OCT-only DR staging. Formally, we compute $q = \mathrm{sim}(\mathbf{z}_{\mathrm{ref}}^{\mathrm{fundus}}, \mathbf{z}_{\mathrm{bridge}}^{\mathrm{fundus}})$ and flag a case for manual review if $q < \tau$.

The practical goal of this rule is not to "improve" the classifier by threshold tuning, but to provide a conservative deployment gate that defers cases where cross-modal projection becomes unreliable due to artifacts or borderline clinical manifestations.

To quantify the sensitivity of the chosen threshold $\tau = 0.8$, we report a local threshold sweep on the discrepant paired hold-out cases (Table S13). At $\tau = 0.8$, all discrepant cases in the paired hold-out set are flagged (5/128, 3.9%), including all examples with severe DR labels (Severe NPDR or PDR) in Table S13.

Lowering the threshold reduces the manual workload but starts missing clinically important under-staging patterns (e.g., Severe NPDR/PDR predicted as a milder stage).

Table S14. Sensitivity of the manual-review threshold τ on the discrepant paired hold-out cases from Table S13. "Flagged" is reported relative to the full paired test set size (128 pairs).

τ	Flagged (n/128)	Flagged (%)	Severe DR among flagged	Interpretation
0.80	5/128	3.9%	3 cases (Severe NPDR or PDR)	Conservative gate; captures all discrepant examples in Table S13.
0.75	4/128	3.1%	3 cases (Severe NPDR or PDR)	Slightly lower workload; misses one borderline boundary case (Mod. → Mild NPDR).
0.70	2/128	1.6%	1 case (PDR)	Lowest workload but misses two severe under-staging examples (Severe NPDR and PDR) shown in Table S13.

Finally, if the prevalence of OCT artifacts increases (motion, low signal, shadowing), the QC score q is expected to decrease more often, which increases the fraction of deferred cases.

This behavior is intentional: under degraded image quality the system shifts from automation to manual verification rather than silently producing potentially unsafe OCT-only DR stages.

The identified issues highlight several critical limitations of the developed system that need attention during clinical implementation.

Dependence on data quality poses a significant risk: motion and lighting artifacts reduce the reliability of the bridge, but standard image quality metrics may not correlate with cosine similarity, necessitating the development of specific quality indicators for cross-modality transfer. The limited field of view of OCT creates a systematic blind spot for peripheral pathologies, such as neovascularization outside the central macular area, leading to underestimation of disease severity when using only the OCT arm of the system.

Borderline clinical cases, such as those involving transitions between DR stages, are marked by inherent uncertainty. Challenges in cross-modality transfer compound this uncertainty, as classification criteria can differ across OCT and fundus modalities. Additionally, the limited sample size of discrepancies, only 5 cases, reduces the analysis's

statistical power and hinders the development of a robust model for predicting bridge failures.

The absence of uncertainty calibration in cosine similarity suggests that a fixed threshold of 0.8 may not be suitable across patient subgroups or conditions. To reduce false alarms while ensuring high sensitivity to actual failures in real-world scenarios, the system should implement adaptive thresholds or probabilistic calibration.

In the analyzed sample, a positive correlation was observed between cosine similarity and the "fundus consistency" metric for bridge predictions, as illustrated by the scatterplot in Figure S1 with a trend of $R^2 \approx 0.406$. This correlation confirms the usefulness of cosine similarity as an indicator of transfer accuracy. This relationship emphasizes that as input data quality and geometric alignment of representations deteriorate, semantic consistency also declines, making artifact and SSI control a critical step.

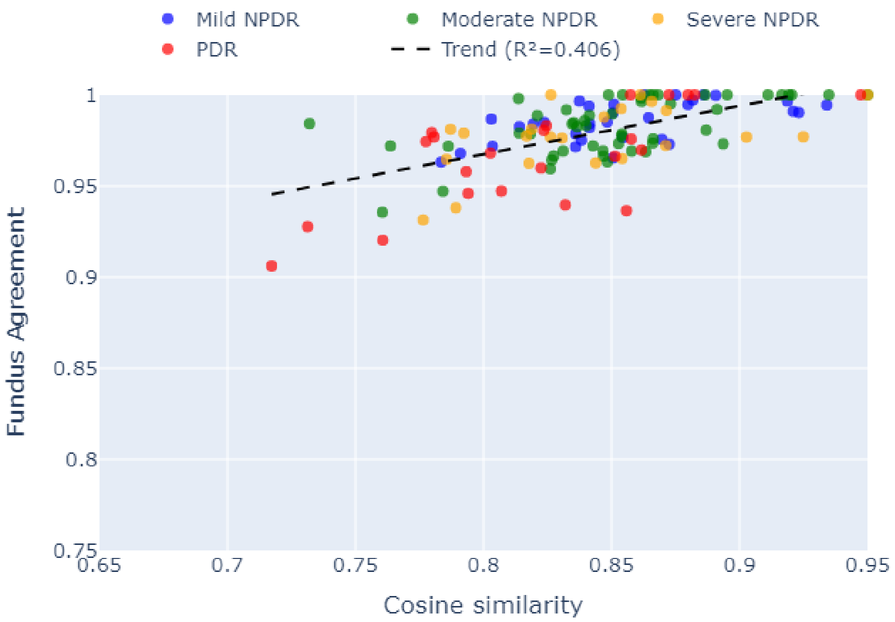

Figure S1. Scatterplot of cosine similarity versus Fundus consistency for the cross-modal bridge for determining DR stages

Section S4.10: Calibration Assessment and Computational Efficiency

To address the distinction between threshold optimization and probability calibration, we emphasize the following separation in our evaluation protocol:

1. **Threshold tuning (F1-optimization)** was performed exclusively on the validation fold, optimizing per-class decision boundaries to maximize the F1-score.
2. **Probability calibration evaluation (ECE, calibration curves)** was performed on the held-out test fold, entirely independent of the threshold selection process.
3. The Expected Calibration Error (ECE) is computed as $\mathrm{ECE} = \sum_i w_i |\mathrm{acc}_i - \mathrm{conf}_i|$, where w_i represents the proportion of samples in the i-th bin, acc_i is the accuracy, and conf_i is the average confidence in that bin.

This separation ensures that the reported high macro-F1 (0.989) is not an artifact of threshold tuning but reflects genuine improvements in both discriminative power and probability quality.

The calibration evaluation of the HMS system is illustrated by the calibration curve in Figure S2. Geometrically, the ECE corresponds to the weighted sum of vertical differences between points on the calibration curve and the diagonal line $y = x$. The system achieves an ECE of $2.1 \pm 0.4\%$, computed via bootstrap with 1000 iterations. This low ECE indicates that the model's predicted probabilities closely align with the actual frequency of correct predictions, ensuring reliability for clinical decision-making [43,44].

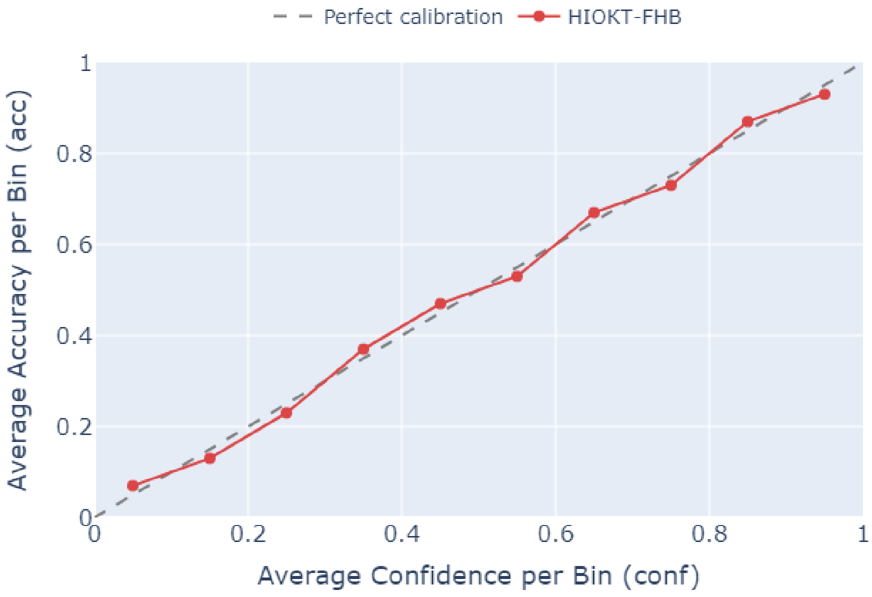

Figure S2. Calibration curve (overall) and per-class ECE contributions. (Curves are computed on held-out test fold using thresholds optimized on validation fold)

Table S15 compares the proposed approach with alternative calibration methods. The results demonstrate that our per-class F1-threshold tuning achieves the lowest ECE (2.1%) while maintaining the highest macro-F1 score, outperforming post-hoc methods like Temperature Scaling and Isotonic Regression on the test set.

Table S15. Comparison of Calibration Methods on Held-Out Test Set. All methods use thresholds optimized on the validation fold only. Confidence intervals (95%) are computed via bootstrap (1000 iterations).

Calibration Method	ECE (%)	ECE 95% CI	Macro-F1	Method Notes
No Calibration	3.2	[2.8–3.6]	0.923	Fixed 0.5 thresholds
**F1-Threshold Tuning (Ours)**	**2.1**	**[1.7–2.5]**	**0.989**	**Per-class F1-optimized**
Temperature Scaling	2.4	[2.0–2.8]	0.968	Post-hoc rescaling
Isotonic Regression	2.2	[1.8–2.6]	0.976	Per-class isotonic fit

0.2. Computational Efficiency

The experiments were conducted using the following hardware configurations: an NVIDIA RTX 3060 GPU and a Ryzen 7 3700X CPU. System optimization studies have confirmed that the efficient implementation of operators and graph optimization can significantly affect both latency and throughput across a variety of CPU and GPU devices. It should be considered when transferring results to other platforms. Table S16 presents the performance metrics measured for each component, indicating that as computational

complexity increases, the inference speed generally decreases, assuming all other factors remain constant.

The modular organization allows tasks to be divided by specialization—such as the parent multi-target model, AMD specialist, fundus model for DR, and the cross-modality OCT-to-fundus bridge. This structure enables independent execution within a pipeline, enhancing portability, interpretability, and scalability across various clinical scenarios. This metric structure aligns with the well-known "speed-accuracy-resources" relationship found in modern convolutional architectures. Increased computational complexity (measured in GFLOPs) and a higher total number of parameters in the "Full Pipeline" naturally lead to lower throughput and higher latency when compared to isolated components. Additionally, using a GPU offers significant improvements over a CPU, highlighting the parallel nature of tensor operations, as confirmed by real-time studies in OCT systems for medical imaging [45].

Table S16. Computational efficiency of system components

Component	GPU (samp/s)	GPU (ms/s)	CPU (samp/s)	CPU (ms/s)	GFLOPs	Params (M)
Parent Model	1139.2	13.7	156.7	97.3	1.82	11.7
AMD Specialist	987.3	15.8	142.1	114.2	1.65	8.4
Fundus Model	1245.8	12.5	178.9	89.6	1.91	11.2
Cross-Modal Bridge	892.5	17.4	98.4	156.8	2.34	5.6
Full Pipeline	634.2	24.6	87.3	187.4	7.72	37.0

The full pipeline processes an OCT volume of 128 B-scans in $\sim$ 3.15 s on the GPU, with an average latency of $\sim$ 24.6 ms per image, meeting real-time imaging requirements and not limiting the clinical workflow's throughput [46].

Section S4.11: Cross-Scanner Validation

Deploying deep learning models in clinical practice requires thorough testing to ensure robustness against variations arising from different equipment types, scanning protocols, and parameter settings. Domain shift, arising from differences in scanner specifications, manufacturers, and signal-processing algorithms, is a crucial factor that can significantly reduce the diagnostic accuracy of models when used across different devices. To evaluate the generalizability of the HMS system across various equipment, we conducted cross-scanner validation using a clinical dataset from the Optimed Laser Vision Restoration Center (Ufa, Russia) comprising 2,185 OCT images.

The cross-domain validation studies shown in Table S17 were conducted in both directions: training on data from one scanner and testing on data from another, which is a standard protocol for assessing cross-device transferability in medical imaging. This design allows us to quantify the degree of performance degradation when encountering feature distributions not present in the training set and to identify the transfer direction with the most significant domain gap [47].

Table S17. Cross-scan validation

Transfer Direction	Acc HMS (%)	HMS AUROC	Acc Eff-B0 (%)	Eff-B0 AUROC	Δ Acc
Avanti XR → REVO NX	86.1 ± 2.3	0.896 ± 0.018	78.8 ± 1.9	0.881 ± 0.014	7.3
REVO NX → Avanti XR	74.7 ± 3.6	0.769 ± 0.020	67.5 ± 2.3	0.628 ± 0.015	7.2

The results demonstrate an asymmetric pattern of cross-scan generalization: transfer from Avanti XR to REVO NX maintains significantly higher accuracy (86.1%) and discriminatory power (AUROC 0.896). In comparison, the reverse direction shows significant

degradation (accuracy 74.7%, AUROC 0.769). This asymmetry is typical for cross-domain tasks in OCT imaging. It is explained by differences in device technical specifications: scanning parameters, noise-reduction algorithms, penetration depth, and resolution vary across manufacturers and models, resulting in uneven domain shifts depending on the transfer direction.

The systematic superiority of HMS over the best-performing baseline method from section 3.10, EfficientNet-B0, by 7.2–7.3 p.p. in both directions indicates improved robustness of the hierarchical architecture to domain variations, consistent with the benefits of multi-stage and adaptive approaches for cross-domain generalization. However, the absolute performance drop from REVO NX to Avanti XR remains significant (a 15.7 p.p. decrease relative to the baseline performance of 90.4% in the within-domain validation), highlighting the algorithm's insufficient robustness to unknown hardware.

The HMS framework showcases the coordinated performance of three components: the parent multi-label OCT model, the AMD staging module, and the fundus-based DR staging model. This collaboration results in clinically relevant patient stratification. The cross-modality bridge between OCT and fundus images aligns the representations from both modalities. Ablation studies confirm the importance of contrast and angular criteria while also revealing typical discrepancies. Probability calibration helps narrow the gap between confidence levels and accuracy, which enhances the reliability of decision-making. The pipeline is computationally efficient enough for near-real-time applications while maintaining reproducibility. Compared to baseline CNNs, it achieves a better balance between discriminability and calibration without compromising robustness. Cross-scan validation demonstrates the system's ability to transfer results between different devices. However, limitations include the availability of paired data, variations in image quality, fixed agreement thresholds, and interdevice bias. These factors underscore the need for adaptive quality control and domain adaptation.

While cross-scanner validation (Table S17) quantifies transfer across devices within the in-house clinical cohort, it does not fully exclude the risk of learning dataset-specific shortcuts when multiple sources are pooled. Therefore, we additionally performed a Leave-One-Source-Out (LOSO) evaluation across the three OCT data sources described in SM Sec. 4.3 (In-house, OCTID, and OCT-Fundus Dataset). In each LOSO setting, we trained the parent OCT multi-label classifier on OCT images from two sources and evaluated it on the held-out third source, using the same preprocessing, architecture, and validation-only threshold tuning protocol as in the main experiments (Table S4).

Table S18 shows that LOSO is a substantially stricter test than mixed-source cross-validation: Macro-F1 decreases by approximately 7.8–12.2% relative to the internal mixed-source baseline (Macro-F1 = 0.989, Table S4). This degradation is expected because LOSO simultaneously introduces multiple shifts: (i) acquisition differences across sites/devices and scanning protocols, (ii) differences in pathology prevalence and stage composition across sources (SM Sec. 4.3, Table SM-2), and (iii) annotation heterogeneity inherent to retrospective multi-source aggregation.

Importantly, despite this stricter setting, performance remains in a clinically meaningful range on an entirely unseen source, indicating that the model does not rely exclusively on source-specific shortcuts. The largest drop is observed when evaluating on the OCT-Fundus Dataset after training on In-house and OCTID (Macro-F1 = 0.868), which is consistent with the fact that this source is enriched in DR/DME-related cases and may differ in acquisition and labeling conventions relative to the in-house AMD-heavy cohort. Conversely, holding out OCTID yields a comparatively smaller degradation (Macro-F1 = 0.912), which is consistent with OCTID acting as a smaller external dataset dominated by coarse disease categories.

Table S18. Leave-One-Source-Out (LOSO) generalization on OCT data. (The parent OCT multi-label model is trained on two sources and evaluated on the held-out third source to test robustness against source-specific shortcuts)

Train Sources	Test Source	Macro-F1	Drop vs Baseline	Interpretation
In-house + OCT-Fundus	OCTID	0.912	-7.8%	Moderate external generalization; residual domain shift
In-house + OCTID	OCT-Fundus	0.868	-12.2%	Stronger domain shift (DR/DME distribution and acquisition differences)
OCTID + OCT-Fundus	In-house	0.881	-10.9%	Stronger domain shift (AMD-heavy clinical cohort and device-specific characteristics)

Overall, LOSO complements the cross-scanner study by providing an explicit source-level external validation proxy. Together, these results support the claim that the hierarchical system learns pathology-driven representations that remain transferable across both devices (Table S17) and sources (Table S18), while also transparently quantifying the residual domain shift that should be expected in real-world deployment.

Section S4.12: AMD–DR Co-activation Audit

To address concerns that the co-occurrence penalty (R_{co}) might artificially suppress true comorbidities, we analyzed the frequency of simultaneous high-confidence predictions ("co-activations") on the test set. A co-activation is defined as an instance where both $p_{\text{AMD}} > \tau_{\text{AMD}}$ (0.15) and $p_{\text{DR}} > \tau_{\text{DR}}$ (0.67).

Table S19 shows that the model retains the capacity to predict both conditions simultaneously, albeit rarely, which reflects the distribution in our training data. The low co-activation rate is consistent with the fact that our dataset consists largely of distinct cohorts (AMD-focused and DR-focused). Importantly, the penalty does not strictly enforce mutual exclusivity but rather discourages low-confidence spurious overlap.

Table S19. Audit of AMD and DR Co-activation Frequency in Test Data

**Metric**	**Value**	**N**	**Interpretation**
Total Test Samples	100%	578	Full held-out set
AMD Prevalence (Predicted)	∼50.0%	289	High prevalence (AMD cohort)
DR Prevalence (Predicted)	∼17.5%	101	DR cohort subset
**Co-activation Rate**	**∼0.9%**	**5**	**Simultaneous detection possible**

Note: Co-activation defined as $p_{AMD} > 0.15$ and $p_{DR} > 0.67$. The non-zero rate confirms that the R_{co} penalty is soft and allows for comorbidity when evidence for both is strong.

Clinical Implication: While the model allows for co-activation, the current R_{co} weight is optimized for datasets with separate labels. In clinical deployment where comorbidity is common (e.g., diabetic patients over 60), we recommend monitoring the "suppression rate" on a calibrated subset and reducing λ_{co} if sensitivity to joint pathology drops.

References

1. M.-L. Zhang and Z.-H. Zhou, “A Review on Multi-Label Learning Algorithms,” *IEEE Trans. Knowl. Data Eng.*, vol. 26, no. 8, pp. 1819–1837, 2014, doi: 10.1109/TKDE.2013.39.
2. T.-Y. Lin, P. Goyal, R. Girshick, K. He and P. Dollár, “Focal Loss for Dense Object Detection,” in *Proceedings of 2017 IEEE International Conference on Computer Vision (ICCV)*, IEEE, 2017, pp. 2999–3007, doi: 10.1109/ICCV.2017.324.
3. Z. C. Lipton, C. Elkan and B. Naryanaswamy, “Optimal Thresholding of Classifiers to Maximize F1 Measure,” in *Machine Learning and Knowledge Discovery in Databases*, vol. 8725, Springer, 2014, pp. 225–239, doi: 10.1007/978-3-662-44851-9_15.
4. S. R. Stahlschmidt, B. Ulfenborg and J. Synnergren, “Multimodal deep learning for biomedical data fusion: a review,” *Briefings in Bioinformatics*, vol. 23, no. 2, p. bbab569, 2022, doi: 10.1093/bib/bbab569.
5. Y. Wen, K. Zhang, Z. Li and Y. Qiao, “A Discriminative Feature Learning Approach for Deep Face Recognition,” in *Computer Vision – ECCV 2016*, vol. 9911, B. Leibe, J. Matas, N. Sebe and M. Welling, Eds., Lecture Notes in Computer Science, Springer, 2016, pp. 499–515, doi: 10.1007/978-3-319-46478-7_31.
6. J. Snell, K. Swersky and R. Zemel, “Prototypical networks for few-shot learning,” *Advances in Neural Information Processing Systems*, vol. 30, 2017, pp. 4077–4087.
7. M. A. Manzoor, S. Albarri, Z. Xian, Z. Meng, P. Nakov and S. Liang, “Multimodality representation learning: a survey on evolution, pretraining and its applications,” *ACM Transactions on Multimedia Computing, Communications and Applications*, vol. 20, no. 1, pp. 1–34, 2023, doi: 10.1145/3596885.
8. T. Silva, A. Marcolini and Y. Dan, “sthalles/SimCLR: Pytorch SimCLR,” Zenodo, Feb. 2021, doi: 10.5281/zenodo.4486327.
9. C. Wan, T. Zhang, Z. Xiong and H. Ye, “Representation Learning for Fault Diagnosis with Contrastive Predictive Coding,” in *Proceedings of 2021 CAA Symposium on Fault Detection, Supervision, and Safety for Technical Processes (SAFEPROCESS)*, IEEE, Dec. 2021, pp. 1–5, doi: 10.1109/SAFEPROCESS52771.2021.9693550.
10. K. He, H. Fan, Y. Wu, S. Xie and R. Girshick, “Momentum Contrast for Unsupervised Visual Representation Learning,” in *Proceedings of 2020 IEEE/CVF Conference on Computer Vision and Pattern Recognition (CVPR)*, IEEE, June 2020, pp. 9726–9735, doi: 10.1109/CVPR42600.2020.00975.
11. T. Chen, S. Kornblith, M. Norouzi and G. Hinton, “A Simple Framework for Contrastive Learning of Visual Representations,” in *Proceedings of the 37th International Conference on Machine Learning*, PMLR, 2020, pp. 1597–1607.
12. A. Radford, J. W. Kim, C. Hallacy, A. Ramesh, G. Goh, S. Agarwal et al., “Learning Transferable Visual Models From Natural Language Supervision,” in *Proceedings of the 38th International Conference on Machine Learning*, PMLR, 2021, pp. 8748–8763.
13. P. Wąż, K. Zorena, A. Murawska and D. Bielińska-Wąż, “Classification Maps: A New Mathematical Tool Supporting the Diagnosis of Age-Related Macular Degeneration,” *J. Personalized Med.*, vol. 13, no. 7, p. 1074, 2023, doi: 10.3390/jpm13071074.
14. “OCTID: Optical Coherence Tomography Image Database,” *Computers & Electrical Engineering*, vol. 81, p. 106532, 2020, doi: 10.1016/j.compeleceng.2019.106532.
15. Translational-Visual-Health-Laboratory, “Translational-Visual-Health-Laboratory/OCT-AND-EYE-FUNDUS-DATASET,” GitHub, [Online]. Available: https://github.com/Translational-Visual-Health-Laboratory/OCT-AND-EYE-FUNDUS-DATASET. [Accessed: Oct. 09, 2025]
16. L. F. Nakayama, R. Oda, P. S. Silva, D. B. Cunha-Vaz, G. Rosa, J. Cunha-Vaz and T. P. Dora, “Diabetic Retinopathy Classification for Supervised Machine Learning Algorithms,” *Int. J. Retina Vitreous*, vol. 8, no. 1, p. 1, 2022, doi: 10.1186/s40942-021-00352-2.
17. L. Cui, C. S. Brun, W. G. Liu, A. Miao, J. Wang, H. Zang et al., “Towards Reliable Healthcare Imaging: A Multifaceted Approach in Class Imbalance Handling for Medical Image Segmentation,” *Interdisciplinary Sciences: Computational Life Sciences*, vol. 17, no. 3, pp. 614–633, 2025, doi: 10.1007/s12539-025-00726-2.
18. “Empirical Investigation of Multi-Source Cross-Validation in Clinical ECG Classification,” *Computers in Biology and Medicine*, vol. 183, p. 109271, 2024, doi: 10.1016/j.compbiomed.2024.109271.
19. M. Kulyabin, A. Nefedova, I. Shilovskiy, D. Vrazhnov, I. Semyachkin, V. Vishnevskiy et al., “OCTDL: Optical Coherence Tomography Dataset for Image-Based Deep Learning Methods,” *Sci. Data*, vol. 11, p. 365, 2024, doi: 10.1038/s41597-024-03182-7.
20. M. K. Bizaki, S. Vassilakopoulos, I. Angelopoulou, A. Koutsouris and D. Fotiadis, “Deep Neural Networks-Based Malignant Breast Lesions Detection and Segmentation from Mammography,” in *Proceedings of 2022 IEEE Nuclear Science Symposium and Medical Imaging Conference (NSS/MIC)*, IEEE, Nov. 2022, pp. 1–3, doi: 10.1109/NSS/MIC44845.2022.10399058.
21. S. Rajaraman, P. Ganesan and S. Antani, “Deep Learning Model Calibration for Improving Performance in Class-Imbalanced Medical Image Classification Tasks,” *PLoS One*, vol. 17, no. 1, p. e0262838, 2022, doi: 10.1371/journal.pone.0262838.
22. S. Kiss, H. S. Chandwani, A. L. Cole, V. D. Patel, O. E. Lunacsek and P. U. Dugel, “Comorbidity and Health Care Visit Burden in Working-Age Commercially Insured Patients with Diabetic Macular Edema,” *Clin. Ophthalmol.*, vol. 10, pp. 2443–2453, 2016, doi: 10.2147/OPTH.S114006.
23. S. Mokhtar, Z. Yusof and H. Sapiri, “Confidence Intervals by Bootstrapping Approach: A Significance Review,” *Malaysian J. Fundamental Appl. Sci.*, vol. 19, no. 1, pp. 30–42, 2023, doi: 10.11113/mjfas.v19n1.2660.

24. P. P and G. R. S, "Detection of Deepfake Medical Images Based on Spatial and Frequency Domain Analysis," in *Proceedings of 2024 IEEE 16th International Conference on Computational Intelligence and Communication Networks (CICN)*, IEEE, Dec. 2024, pp. 611–617, doi: 10.1109/CICN63059.2024.10847427.
25. C. Lobo, J. Teixeira, P. Marques, R. Freitas, D. Castanho, D. Martins et al., "Characterisation of Progression of Macular Oedema in the Initial Stages of Diabetic Retinopathy: A 3-Year Longitudinal Study," *Eye*, vol. 37, no. 2, pp. 313–319, 2023, doi: 10.1038/s41433-022-01937-3.
26. C. Toma, R. Pop-Moldovan, E. Birkás, A. Bursuc, F. Simo-Stela, V. P. M. Breeze et al., "Microvascular Changes in Eyes with Non-Proliferative Diabetic Retinopathy With or Without Macular Microaneurysms: An OCT-Angiography Study," *Acta Diabetol.*, vol. 62, no. 5, pp. 753–761, 2025, doi: 10.1007/s00592-024-02394-y.
27. M. L. Enzendorfer, M. Tratnig-Frankl, A. Eidenberger, J. Schrittwieser, L. Kuchernig and U. Schmidt-Erfurth, "Rethinking Clinical Trials in Age-Related Macular Degeneration: How AI-Based OCT Analysis Can Support Successful Outcomes," *Pharmaceuticals*, vol. 18, no. 3, p. 284, 2025, doi: 10.3390/ph18030284.
28. L. Wu, P. Fernandez-Loaiza, J. Sauma, E. Hernandez-Bogantes and M. Masis, "Classification of Diabetic Retinopathy and Diabetic Macular Edema," *World J. Diabetes*, vol. 4, no. 6, pp. 290–294, 2013, doi: 10.4239/wjd.v4.i6.290.
29. S. T. Kaderli, A. Karalezli, C. Kaya, S. Korkmaz and S. Sul, "Sensitivity and Specificity of the Optical Coherence Tomography Angiography for Detection of Neovascularization and Evaluation of Peripheral Ischemia in Diabetic Retinopathy," *Beyoglu Eye J.*, vol. 7, no. 4, p. 273, 2022.
30. C. P. Wilkinson, F. L. Ferris, R. E. Klein, P. P. Lee, A. D. Agardh, M. Davis et al., "Proposed International Clinical Diabetic Retinopathy and Diabetic Macular Edema Disease Severity Scales," *Ophthalmology*, vol. 110, no. 9, pp. 1677–1682, 2003, doi: 10.1016/S0161-6420(03)00475-5.
31. Early Treatment Diabetic Retinopathy Study Research Group, "Grading Diabetic Retinopathy from Stereoscopic Color Fundus Photographs: An Extension of the Modified Airlie House Classification," *Ophthalmology*, vol. 98, no. 5, pp. 786–806, 1991, doi: 10.1016/S0161-6420(13)38015-7.
32. Y. Bi, J. Xie and H. Wang, "Contrastive Learning-Based Feature Modulation Strategy for Test-Time Adaptation in Medical Image Segmentation," in *Proceedings of 2025 28th International Conference on Computer Supported Cooperative Work in Design (CSCWD)*, IEEE, May 2025, pp. 916–921, doi: 10.1109/CSCWD64889.2025.11033539.
33. E. Sükei, I. Frey, K. Gawish, B. Neumann, U. Kuch, A. Stöhr et al., "Multi-Modal Representation Learning in Retinal Imaging Using Self-Supervised Learning for Enhanced Clinical Predictions," *Sci. Rep.*, vol. 14, p. 26802, 2024, doi: 10.1038/s41598-024-78515-y.
34. S. Rajaraman, G. Zamzmi and S. K. Antani, "Novel Loss Functions for Ensemble-Based Medical Image Classification," *PLoS One*, vol. 16, no. 12, p. e0261307, 2021, doi: 10.1371/journal.pone.0261307.
35. R. Viñals and J.-P. Thiran, "A KL Divergence-Based Loss for In Vivo Ultrafast Ultrasound Image Enhancement With Deep Learning," *J. Imaging*, vol. 9, no. 12, p. 256, 2023, doi: 10.3390/jimaging9120256.
36. X. Lei, J. Chen, Z. J. Zhang, B. Lin, A. S. Fawzi, N. Voigt et al., "A Cross-Modal Feature Fusion Method to Diagnose Macular Fibrosis in Neovascular Age-Related Macular Degeneration," in *Proceedings of 2024 IEEE International Symposium on Biomedical Imaging (ISBI)*, IEEE, May 2024, pp. 1–5, doi: 10.1109/ISBI56570.2024.10635126.
37. S. Mo, Z. Sun and C. Li, "Rethinking Prototypical Contrastive Learning Through Alignment, Uniformity and Correlation," *Neurocomputing*, vol. 506, pp. 244–256, 2022, doi: 10.1016/j.neucom.2022.07.060.
38. A. Zedadra, M. Y. Salah-Salah, O. Zedadra and A. Guerrieri, "Multi-Modal AI for Multi-Label Retinal Disease Prediction Using OCT and Fundus Images: A Hybrid Approach," *Sensors*, vol. 25, no. 14, p. 4492, 2025, doi: 10.3390/s25144492.
39. L. A. Hendricks, J. Mellor, R. Schneider, J.-B. Alayrac and A. Nematzadeh, "Decoupling the Role of Data, Attention, and Losses in Multimodal Transformers," *Transactions of the Association for Computational Linguistics*, vol. 9, pp. 570–585, 2021, doi: 10.1162/tacl_a_00393.
40. J. Li, R. Selvaraju, A. Gotmare, S. Joty, C. Xiong and S. C. H. Hoi, "Align Before Fuse: Vision and Language Representation Learning With Momentum Distillation," in *Advances in Neural Information Processing Systems*, vol. 34, pp. 9694–9705, 2021.
41. M. Jiang, Z. Wang, J. Kong and D. Zhuang, "MCFusion: Infrared and Visible Image Fusion Based Multiscale Receptive Field and Cross-Modal Enhanced Attention Mechanism," *J. Electronic Imaging*, vol. 33, no. 1, p. 013039, 2024, doi: 10.1117/1.JEI.33.1.013039.
42. Y. Zhu, J. Ai, W. Xue, Z. Wang and Z. Zhao, "Cross-Modal Ship Detection From Optical to SAR Images Based on Pixel- and Feature-Level Progressive Transfer," *IEEE Sensors J.*, vol. 25, no. 8, pp. 13344–13356, 2025, doi: 10.1109/JSEN.2025.3543520.
43. G. Liang, Y. Zhang and N. Jacobs, "Neural Network Calibration for Medical Imaging Classification Using DCA Regularization," in *Proceedings of the 2019 IEEE International Conference on Image Processing (ICIP)*, IEEE, Sept. 2019, pp. 335–339, doi: 10.1109/ICIP.2019.8803142.
44. A. Chanda, K. Choudhury, S. Roy, S. Biswas and S. Kuiry, "Evaluating Temperature Scaling Calibration Effectiveness for CNNs Under Varying Noise Levels in Brain Tumour Detection," *IEEE Access*, vol. 13, pp. 108256–108272, 2025, doi: 10.1109/ACCESS.2025.3528412.

45. M. Li, Y. Jiang, Y. Zhang and H. Zhu, "Medical Image Analysis Using Deep Learning Algorithms," *Front. Public Health*, vol. 11, p. 1273253, 2023, doi: 10.3389/fpubh.2023.1273253.
46. Q. Chen, M. M. K. Sarhan, C.-L. Chen, Y. A. Müller, L. Schmetterer, C. M. Svensson et al., "AI Workflow, External Validation, and Development in Eye Disease Diagnosis," *JAMA Netw. Open*, vol. 8, no. 7, p. e2517204, 2025, doi: 10.1001/jamanetworkopen.2025.17204.
47. H. Nouri, R. Nasri and S.-H. Abtahi, "Addressing Inter-Device Variations in Optical Coherence Tomography Angiography: Will Image-to-Image Translation Systems Help?," *Int. J. Retina Vitreous*, vol. 9, p. 51, 2023, doi: 10.1186/s40942-023-00491-8.

Disclaimer/Publisher's Note: The statements, opinions and data contained in all publications are solely those of the individual author(s) and contributor(s) and not of MDPI and/or the editor(s). MDPI and/or the editor(s) disclaim responsibility for any injury to people or property resulting from any ideas, methods, instructions or products referred to in the content.
