# Supplementary figures and images for: A Hierarchical Deep Learning Architecture for Diagnosing Retinal Diseases Using Cross-Modal OCT to Fundus Translation in the Lack of Paired Data
Source: J Imaging. 2026 Jan 8;12(1):36. doi: 10.3390/jimaging12010036 (PMC12842718; doi:10.3390/jimaging12010036)

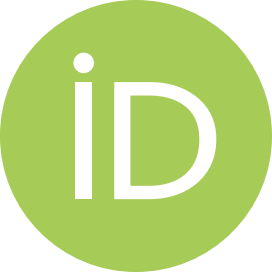

Supplement: Supplementary file 1 [file jimaging-12-00036-s001.zip › jimaging-4042548-supplementary/Definitions/logo-orcid.pdf]

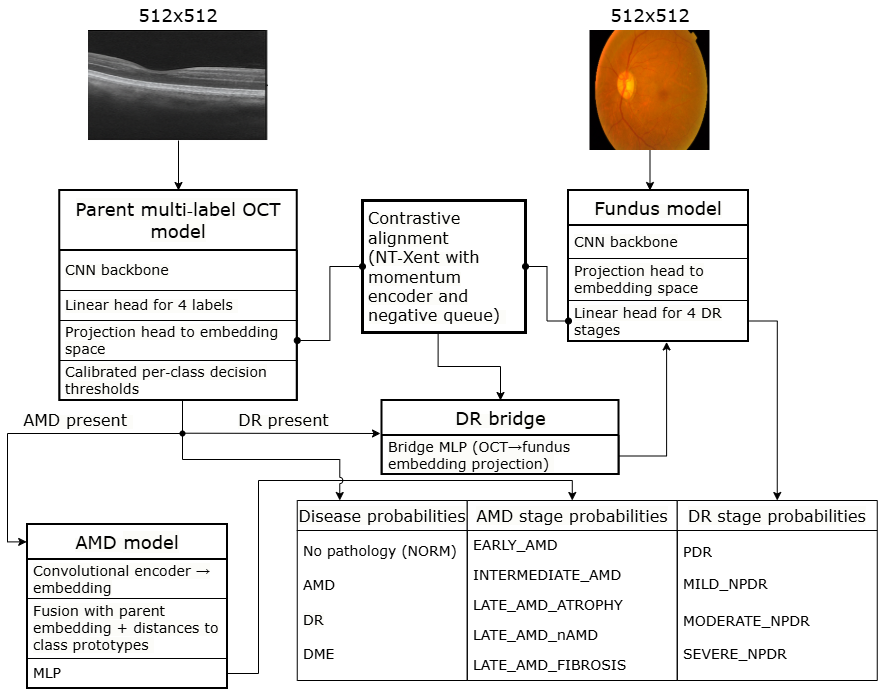

Supplement: Supplementary file 1 [file jimaging-12-00036-s001.zip › jimaging-4042548-supplementary/fig1.png]

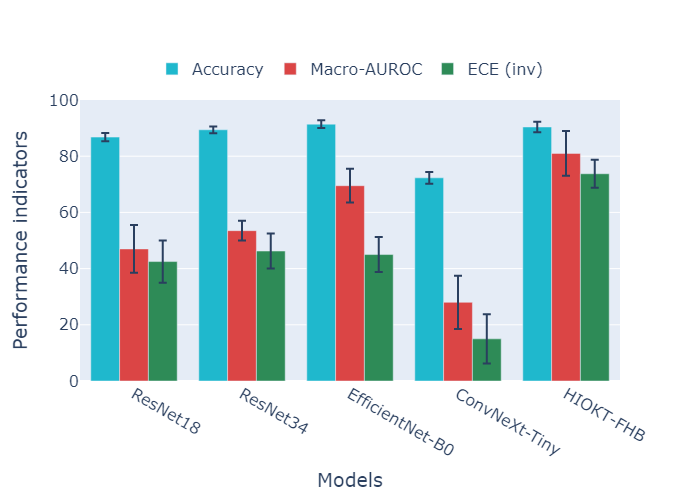

Supplement: Supplementary file 1 [file jimaging-12-00036-s001.zip › jimaging-4042548-supplementary/fig2.png]

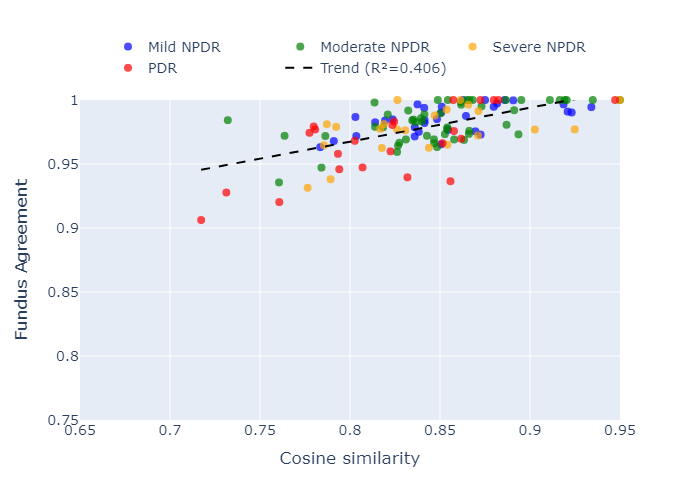

Supplement: Supplementary file 1 [file jimaging-12-00036-s001.zip › jimaging-4042548-supplementary/fig3.png]

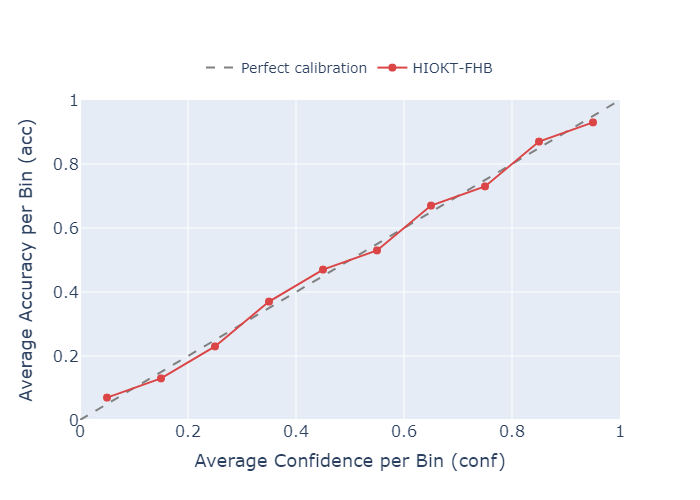

Supplement: Supplementary file 1 [file jimaging-12-00036-s001.zip › jimaging-4042548-supplementary/fig4.png]
